# Stress‐Induced Activation of Prolactin‐NR4A1‐Midkine Axis Exacerbates Skin Inflammation

**DOI:** 10.1002/advs.202509679

**Published:** 2025-11-05

**Authors:** Zhiguo Li, Huiyi Quan, Wanting Liu, Jiaoling Chen, Mengyang Chu, Xin Tang, Ke Xue, Xuan Liu, Jingyi Ma, Yaxing Bai, Ruina Dong, Bing Li, Junfeng Hao, Wei Guo, Qingyang Li, Erle Dang, Johann E Gudjonsson, Gang Wang, Shuai Shao

**Affiliations:** ^1^ Department of Dermatology Xijing Hospital Fourth Military Medical University Xi'an Shaanxi 710032 China; ^2^ Department of Dermatology University of Michigan Ann Arbor MI 48109 USA

**Keywords:** fibroblasts, midkine, NR4A1, prolactin, stress

## Abstract

Stress is an established trigger of skin inflammation and disease flares; however, the mechanisms have remained unclear. Here, using human data, mechanistic exploration, and single‐cell RNA sequencing in mouse models of skin inflammation under stress challenge, prolactin is identified as a key mediator linking stress to inflammatory responses in the skin through an NR4A1‐midkine axis in *APCDD1* ^+^ fibroblasts in the upper dermis. The data shows that prolactin is increased in the plasma of psoriasis patients with high levels of stress and in stressed mice, which activates transcription factor NR4A1 in *APCDD1* ^+^ fibroblasts, promoting secretion of midkine, and amplification of immune infiltration and responses in neighboring cells. Targeting NR4A1 or midkine effectively reverses these inflammatory effects in vivo. These data provide a novel mechanism for stress‐related amplification of skin inflammation and identify NR4A1 and midkine as potential therapeutic targets to mitigate stress‐exacerbated inflammation.

## Graphical abstract

Chronic psychological stress activates prolactin signaling to reprogram dermal fibroblasts into *APCDD1* ^+^ inflammatory effectors via NR4A1, driving midkine secretion. This paracrine hub amplifies keratinocyte proliferation and immune recruitment, thus exacerbating skin inflammation. Therapeutic inhibition of the NR4A1‐midkine axis not only neutralizes stress‐induced inflammation but nominates *APCDD1* ^+^ fibroblasts as the previously unrecognized targets for intercepting neuroendocrine‐immune circuit.

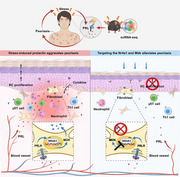

## 1 Introduction

Stress is a natural response that can be physical or emotional and has been associated with exacerbation of various conditions, including inflammation^[^ ^1^, ^2^, ^3^ ^]^ and cancer.^[^ ^4^, ^5^ ^]^ We have recently demonstrated a causal connection between stress‐related states, such as major depressive disorders and anxiety, and an increased risk of psoriasis.^[^ ^6^ ^]^ The use of antidepressants, which can alleviate or eliminate many inflammatory symptoms, further underscores the connection between stress‐induced behavioral changes and chronic inflammation.^[^ ^7^ ^]^ However, the mechanisms involved have remained mostly unknown.

Stress has been shown to impact peripheral immunity through activation of the hypothalamic‐pituitary‐adrenal (HPA) axis and sympathetic adrenal‐medullary (SAM), including both the sympathetic and parasympathetic systems.^[^ ^8^, ^9^ ^]^ Under stress, the SAM and HPA axes promote the secretion of catecholamines and glucocorticoids that affect cells throughout the body.^[^ ^10^ ^]^ Prolactin, a neuroendocrine peptide produced by the anterior pituitary, plays a significant role in immune modulation through cytokine and hematopoietic receptor signaling.^[^ ^11^ ^]^ Prolactin influences the functional states of adaptive immune cells, such as T and B lymphocytes, and innate immune cells like macrophages.^[^ ^11^, ^12^ ^]^ Additionally, other hormones and mediators, including growth hormone^[^ ^13^ ^]^ and thyroid hormones,^[^ ^14^ ^]^ also play a role in the stress responses and may impact skin function, though the mechanisms remain poorly understood. Importantly, a better understanding of how stress induces and exacerbates skin inflammation is essential for identifying at‐risk individuals and developing effective prevention or therapeutic strategies.

In this study, we evaluated anxiety and depression in psoriasis patients and measured serum hormone levels under varying stress conditions. Moreover, we used a validated murine model of chronic restraint stress (CRS), which induces sustained anxiety and depression‐like behaviors, to assess the impact of stress on skin immunity under an imiquimod (IMQ) challenge. Through single‐cell sequencing of skin lesions, we examined how chronic stress affects skin immunity and discovered that stress alters fibroblast heterogeneity through prolactin signaling. Further investigations, including cell experiments, RNA‐seq, and chromatin immunoprecipitation, revealed that nuclear receptor 4A1 (NR4A1) plays a key role in fibroblast responses under stress. Blocking NR4A1 or midkine alleviated the stress‐induced exacerbation of skin inflammation. These findings highlight potential therapeutic targets for managing stress‐associated skin inflammation.

## 2 Results

### 2.1 Stress Exacerbates Skin Inflammation in Humans and is Associated with Increased Serum Levels of Prolactin

**Figure 1:**
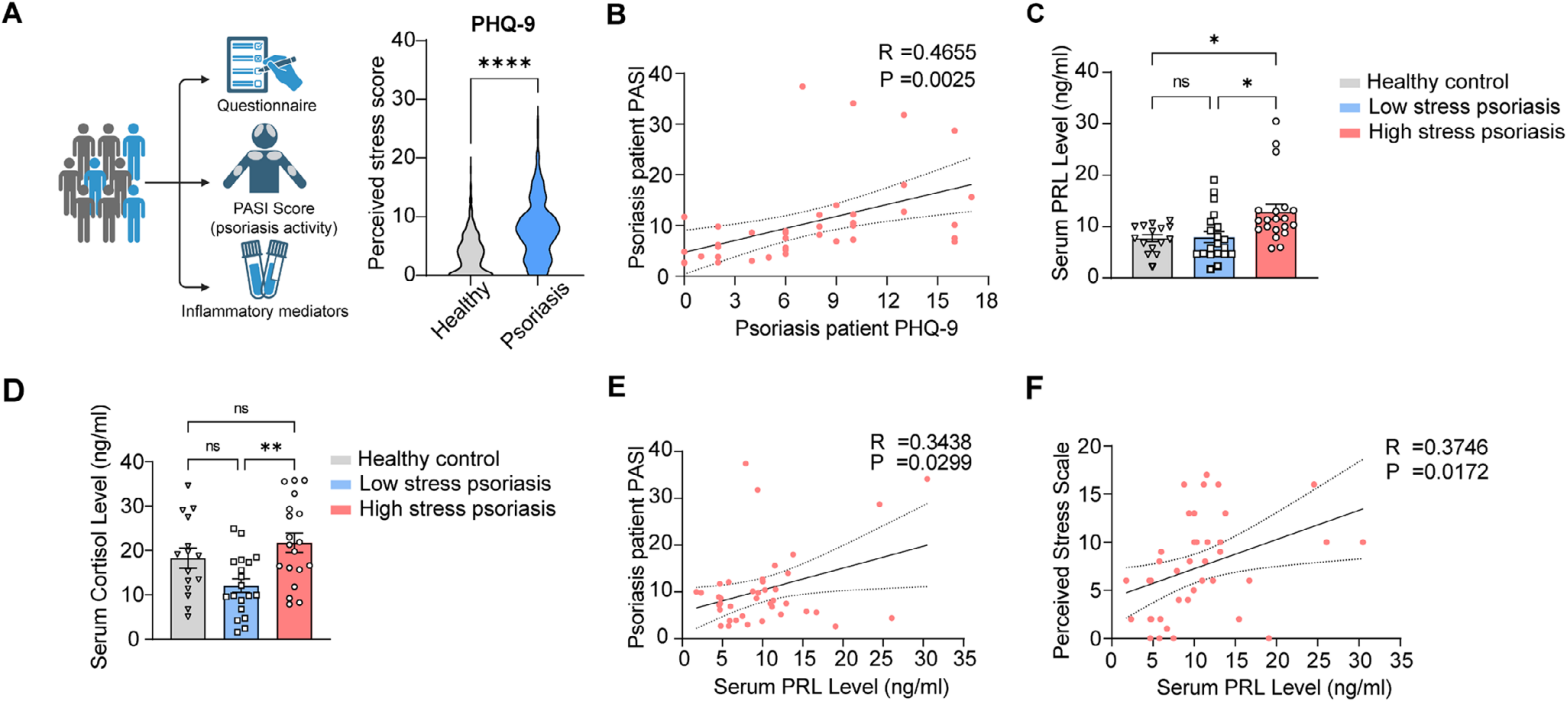
Psychological stress exacerbates skin inflammation in humans. A) Left: Schematic representation of a real‐world study assessing the impact of stress on psoriasis patients. Right: Stress scores of psoriasis patients (*n* = 211) vs healthy controls (*n* = 239). B) Pearson's correlation of questionnaire‐based stress scores and PASI. C,D) Serum levels of PRL (C) or cortisol (D) in control (*n* = 15), unstressed (*n* = 20), and stressed psoriasis patients (*n* = 20). E) Pearson's correlation of serum PRL level and disease severity (PASI). F) Pearson's correlation of serum PRL level and questionnaire‐based stress scores. Plotted are means±SD. ^*^ *p* <0.05, ^**^ *p* < 0.01, ^****^ *p* < 0.0001, ns, not significant (A: unpaired Student's *t*‐test, C, D: one‐way ANOVA with Tukey's multiple comparisons test). PASI: Psoriasis Area Severity Index; PHQ‐9: Patient Health Questionnaire‐9; PRL: prolactin.

To explore the association of chronic stress with skin inflammation, we conducted Patient Health Questionnaire‐9 (PHQ‐9) assessments^[^ ^15^ ^]^ on psoriasis patients (*n* = 211) and healthy controls (*n* = 239) (Table S1, Supporting Information). Compared to healthy controls (PHQ‐9 mean ± SD: 3.7 ± 3.4), psoriasis patients reported an increased stress perception (PHQ‐9 mean ± SD: 7.8 ± 5.0, **Figure** **1**A). Notably, there was a positive correlation between psoriasis severity (PASI) and PHQ‐9 scores (*R* = 0.4655, *P* < 0.01, Figure 1B), further suggesting that stress may promote increased inflammatory activity.

To further dissect the relationship between immune response and stress, psoriasis patients were divided into a high‐stress group (PHQ‐9 ≥ 7) and a low‐stress group (PHQ‐9 < 7) (Table S2, Supporting Information). The levels of hormones associated with stress response in all participants were assessed by ELISA, including prolactin (PRL), estradiol (E2), thyroid‐stimulating hormone (TSH), growth hormone (GH), and cortisol. Notably, psoriasis patients with high‐stress exhibited significantly elevated serum PRL compared to low‐stress patients and healthy controls (Figure 1C). Similarly, serum cortisol level was higher in the high‐stress than that in the low‐stress psoriasis groups, though not exceeding the level observed in the control groups (Figure 1D). TSH level was significantly lower in psoriasis patients than that in healthy controls, regardless of their PHQ‐9 scores, whereas mean serum E2 and GH levels did not differ among the three groups (Figure S1A, Supporting Information). Furthermore, we identified a significant, though not strong, association of serum PRL with both PASI (*R* = 0.3438, *P* < 0.05) and PHQ‐9 scores (*R* = 0.3746, *P* < 0.05) (Figure 1E,F), while cortisol levels showed no association with disease severity (*P* > 0.05) (Figure S1B, Supporting Information). Building on these findings and prior research on the effects of prolactin in mental stress,^[^ ^16^, ^17^, ^18^ ^]^ we further validate the link between stress and exacerbated skin inflammation, highlighting PRL as a key neuroendocrine mediator in this process.

### 2.2 Stress Aggravates IMQ‐Induced Skin Inflammation

**Figure 2:**
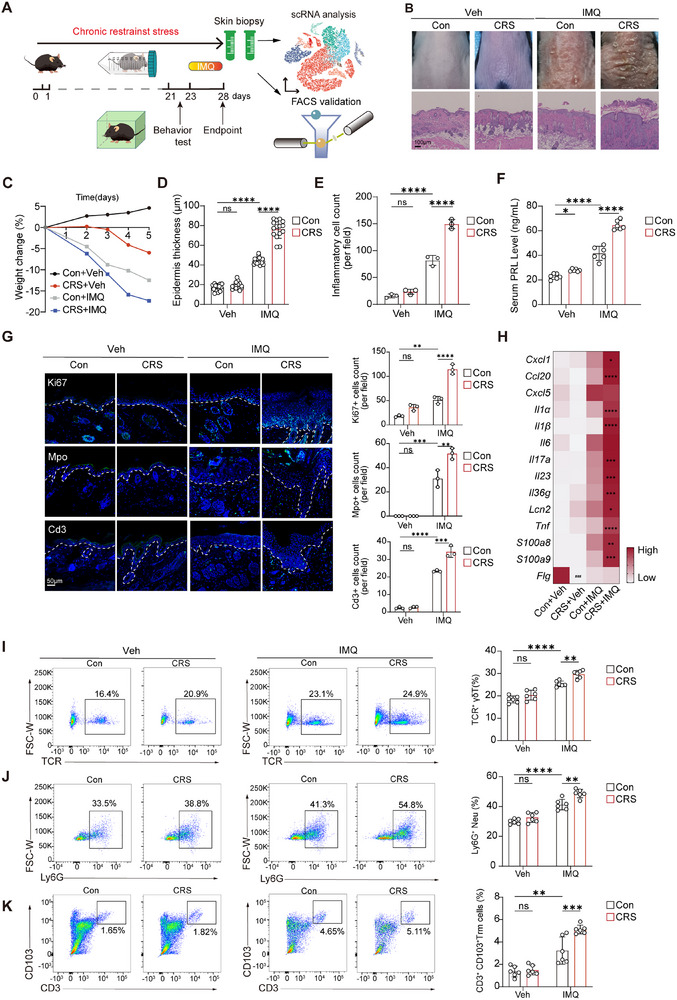
Psychological stress exacerbates psoriasiform dermatitis and can be alleviated by antidepressants. A) Schematic of experimental stress‐skin inflammation paradigm. B) Representative macroscopic views (upper panel) and H&E staining (lower panel) of mouse back skin. Scale bar, 100 µm. C) Weight changes across several skin conditions (*n* = 3). D, E) Epidemal thickness D) and infiltrated inflammatory cell number E) across several skin conditions (*n* = 3). F) The serum levels of PRL of mice in each group (*n* = 6). G) Representative IF and quantitation of Ki67^+^ epidermal cells, Mpo^+^ neutrophils, and Cd3^+^ T cells in mouse skin (*n* = 3). Scale bar, 50 µm. H) Transcriptional levels of inflammatory genes in murine skin (*n* = 3). The hashtag denotes the *P* value from the comparison between the stressed mice and the control group, whereas the asterisk represents the *P* value from the comparison of IMQ‐challenged mice with and without stress. I–K) FCM analysis of lesional γδT cells (I), neutrophils (J) and tissue‐resident memory T cells (K) to validate the effect of chronic psychological stress on skin inflammation (*n* = 6). All results are shown as the means ± SD. ^*^ *p* < 0.05, ^**^ *p* < 0.01, ^***/###^ *p* < 0.001, ^****^ *p* < 0.0001, ns, not significant (D–K: two‐way ANOVA). Con: control; CRS: chronic restraint stress; FACS: fluorescence activated cell sorting; FCM: flow cytometry; IMQ: imiquimod; Neu: neutrophil; PASI: Psoriasis Area Severity Index; PRL: prolactin; scRNA: Single‐cell RNA sequencing; Trm: tissue‐resident memory T cells; Veh: vehicle.

To investigate the impact of stress on skin inflammation in vivo, we employed a chronic restraint stress (CRS) mouse model^[^ ^19^ ^]^ and induced skin inflammation with topical imiquimod (IMQ) (**Figure** **2**A, four experimental groups: Con+Vehicle, CRS+Vehicle, Con+IMQ, CRS+IMQ). Stressed mice exhibited depressive behaviors, as indicated by reduced locomotion and open‐field test results (Figure S2A–D, Supporting Information). We observed amplified skin inflammation in stressed mice treated with IMQ (Figure 2B), characterized by greater weight loss (Figure 2C), increased thickening and scaling of the epidermis (Figure 2D), erythema, and increased immune cell infiltration (Figure 2E), compared to non‐stressed IMQ and control mice (*P* < 0.01). Consistent with human data (Figure 1C), stressed IMQ‐treated mice exhibited significantly elevated circulating prolactin level (Figure 2F). Notably, immunofluorescence (IF) analyses revealed a marked expansion of proliferating keratinocytes (Ki67^+^), accompanied by increased T‐cell infiltration (Cd3^+^) in both the epidermal and dermal compartments, as well as (Mpo^+^) neutrophil accumulation predominantly forming epidermal microabscesses (Figure 2G; Figure S2E, Supporting Information). Stress also upregulated the expression of proinflammatory cytokines including *Il17a, Il23, Tnf, Il36g, Il1α, Il1β*, chemokines including *Cxcl1, Ccl20*, as well as antimicrobial peptides such as *S100a8, S100a9, Lcn2* (Figure 2H), further amplifying IMQ‐driven cutaneous inflammation. Flow cytometry (FCM) corroborated these findings, confirming the enhanced immune infiltration induced by stress (Figure 2I–K; Figure S2F,G, Supporting Information).

To confirm the impact of stress on the inflammatory phenotype, we treated the mice with an intraperitoneal (*i.p*.) injection of agomelatine (Figure S2H, Supporting Information), which has been shown to alleviate stress‐induced physiological changes and depression.^[^ ^20^ ^]^ Notably, treatment with agomelatine in stressed IMQ mice resulted in decreased scaling and thinner epidermis and reduced infiltration of Cd3^+^ T cells and Mpo^+^ neutrophils (Figure S2I, Supporting Information). Previously validated proinflammatory markers (e.g., *Il1α, Il1β, Il17a, Il23, Tnf, Il36g, Cxcl1*) were also reduced following agomelatine treatment (Figure S2J, Supporting Information). These findings suggest that stress alone may not induce visible erythema and scales, but predisposes to a proinflammatory state in the skin.

### 2.3 Stress Promotes a Proinflammatory Microenvironment and Reprograms Fibroblasts in the Skin

**Figure 3:**
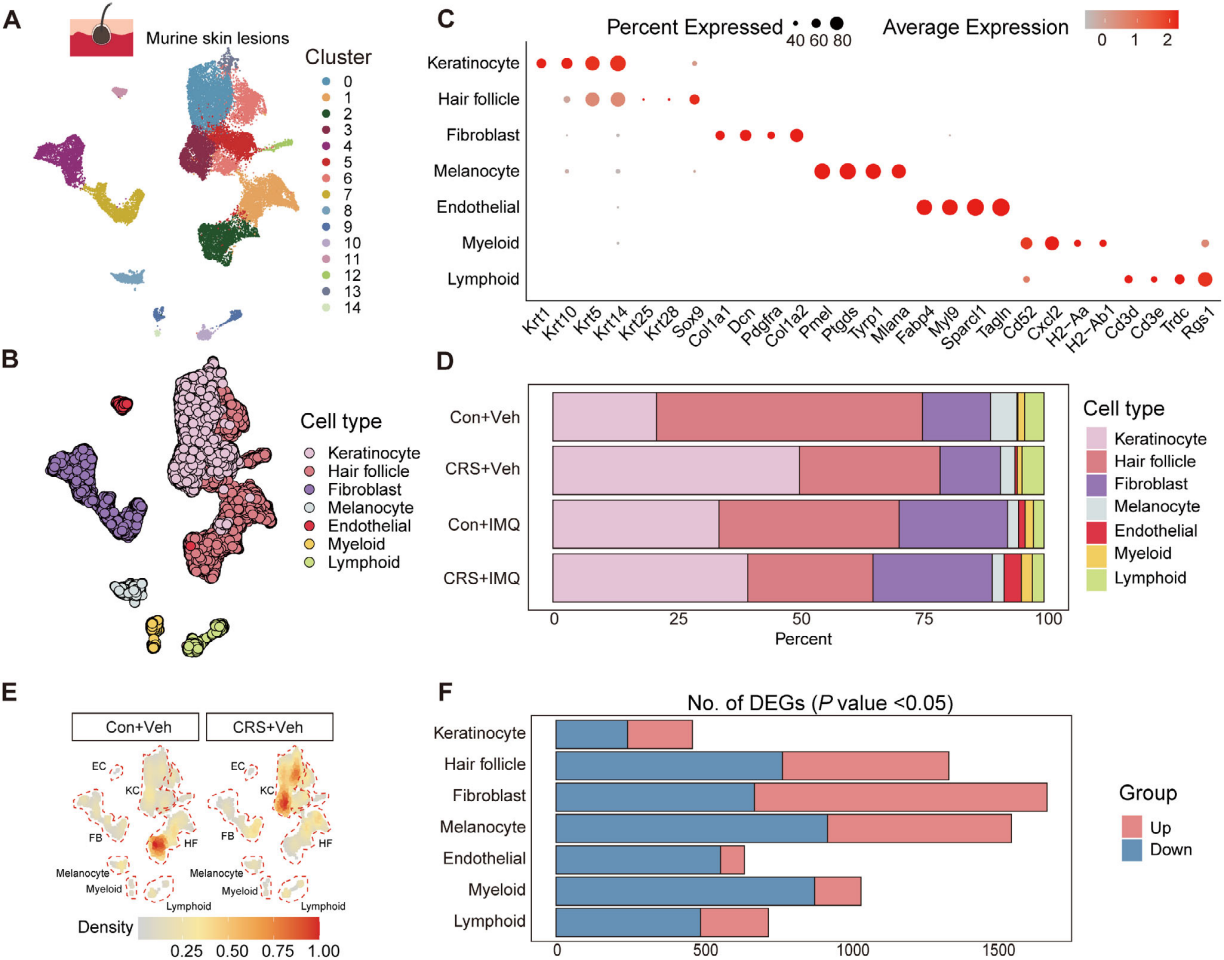
Stress alters skin keratinocytes, fibroblasts, and immune compositions. A) UMAP representation of 26606 skin cells from distinct skin conditions. B) UMAP plot of skin cells colored by cell types. C) Dot plots of representative marker genes from distinct cell compartments across several skin states. Dot color represents average expression. Dot size represents percent expression. The fraction of cells at which to draw the smallest dot was set as 25%. D) Bar chart of the distribution of cell clusters in skin tissues of mice described in A. E) UMAP view of cluster density displaying skin cell distribution across experimental groups. Higher relative cell density is shown as bright orange. F) Number of DEGs in the individual cell types. Con: control; CRS: chronic restraint stress; DEGs: differentially expressed genes; EC: endothelial cell; FB: fibroblast; HF: hair follicle; IMQ: imiquimod; KC: keratinocyte; UMAP: uniform manifold approximation and projection; Veh: vehicle.

To investigate the mechanism by which chronic stress promotes increased inflammation in the skin, we performed single‐cell RNA sequencing (scRNA‐seq) of skin biopsies from each of the four experimental groups (Con+Vehicle, CRS+Vehicle, Con+IMQ, CRS+IMQ). This yielded a single‐cell atlas with 23983 genes and 26606 cells in 15 distinct cell clusters (**Figure** **3**A). The resulting cell clusters were annotated and assigned to seven cell types by matching the cluster signatures with known lineage markers (Figure 3B,C; Table S3, Supporting Information). Cell abundance analysis demonstrated that relative proportion of keratinocytes and endothelial cells was elevated in stressed IMQ mice compared to unstressed IMQ‐treated mice, along with moderate increases in fibroblasts and myeloid cells (Figure 3D). Notably, relative abundance of keratinocytes and lymphoid cells exhibited a marked expansion in stressed mice compared to untreated controls (Figure 3D), despite the absence of overt inflammation (Figure 2G–K).

We first focused on keratinocytes, as they displayed a notable increase in relative abundance under stress conditions (Figure 3D,E; Figure S3A, Supporting Information). According to distinct keratinocyte differentiation states, keratinocytes were further divided into 4 classical subpopulations, including undifferentiated (*Krt5, Krt14*), proliferative (*Mki67, Top2a*), differentiating (*Krt1, Krt10*), and differentiated states (*Lor, Krt2*) (Figure S3B,C, Supporting Information). Subcluster analysis and density mapping revealed an accumulation of hyperproliferative keratinocyte states specifically in CRS+IMQ mice (Figure S3A,D, Supporting Information), whereas CRS+Vehicle mice did not exhibit this proliferative shift (Figure S3D, Supporting Information). To assess whether proliferating keratinocytes contribute functionally to stress‐aggravated inflammation, we performed pseudotime trajectory analysis. Keratinocytes from stressed mice aligned along a differentiation continuum, progressing from undifferentiated (*Krt5, Krt14*) through proliferative (*Mki67, Top2a*) to differentiating (*Krt1, Krt10*) and terminally differentiated states (*Lor, Krt2*), closely mirroring the trajectory observed in IMQ‐treated mice (Figure S3E, Supporting Information). In contrast, several lines of evidence argue against a proliferative response in CRS+Vehicle mice: histological analysis (H&E) showed no changes (Figure 2B,D), Ki67 staining was absent (Figure 2G), and transcriptomic differences relative to Con+Vehicle were minimal (Figure 3F; Table S5, Supporting Information). Importantly, although IMQ‐treated mice displayed a lower proportion of keratinocytes compared with CRS+Veh (Figure 3D), histological quantification confirmed an increase in their absolute numbers (Figure 2B,D). Thus, the higher keratinocyte proportion observed in stressed mice likely reflects reduced hair follicle activity, a well‐documented consequence of chronic stress,^[^ ^21^ ^]^ rather than true keratinocyte expansion. This highlights the need for cautious interpretation of single‐cell compositional data, as relative changes may reflect redistribution rather than absolute cell number alterations. Accordingly, the absolute numbers of each cell type across groups were shown in the Supplementary Tables (Table S4, Supporting Information).

We next interrogated immune cells, and subtype analysis revealed a mild increase in T‐cell proportions in stressed skin, alongside elevated neutrophil counts amplified by IMQ (Figure 3D,E; Figure S3F–I, Supporting Information). These findings were corroborated by IF and FCM (Figure 2G,I–K; Figure S2F,G, Supporting Information). However, differential expression analysis revealed limited transcriptional changes within lymphoid or myeloid subsets between stressed and unstressed groups (Figure 3F; Table S5, Supporting Information), suggesting that immune cells act as downstream amplifiers rather than primary mediators of stress‐induced cutaneous pathology.

Then we focused on stromal cells, particularly fibroblasts, which exhibited pronounced transcriptional remodeling under chronic stress (Figure 3F; Table S5, Supporting Information). Cell composition analysis demonstrated a marked increase in fibroblast numbers in CRS+IMQ skin (Figure 3D; Table S4, Supporting Information), implicating synergistic effects of stress and inflammation in driving fibroblast expansion. Notably, in CRS+Vehicle group, fibroblast density shifted spatially (Figure 3E) without significant proportional changes, indicating that stress primarily reprograms fibroblast identity and function rather than simply expanding their pool.

Hair follicles and melanocytes exhibited the highest number of stress‐associated differentially expressed genes (DEGs) (Figure 3F; Table S5, Supporting Information), but their low abundance argue against a central role in stress‐driven skin inflammation (Figure 3D). Endothelial cells increased progressively from control to stressed inflamed skin, yet displayed few DEGs (Figure 3F). Gene set enrichment indicated activation of angiogenesis rather than neuroendocrine–immune pathways (Figure S3J,K, Supporting Information), suggesting endothelial cells act as downstream responders rather than initiators of stress‐mediated pathology.

Collectively, these findings identify fibroblasts as key transcriptionally responsive stromal cells in stressed skin, suggesting they function as pivotal effectors linking psychological stress to a proinflammatory cutaneous microenvironment.

### 2.4 Stress‐Exacerbated Skin Inflammation is Associated with Activation of Dermal Fibroblasts Through Prolactin Receptor Signaling

**Figure 4:**
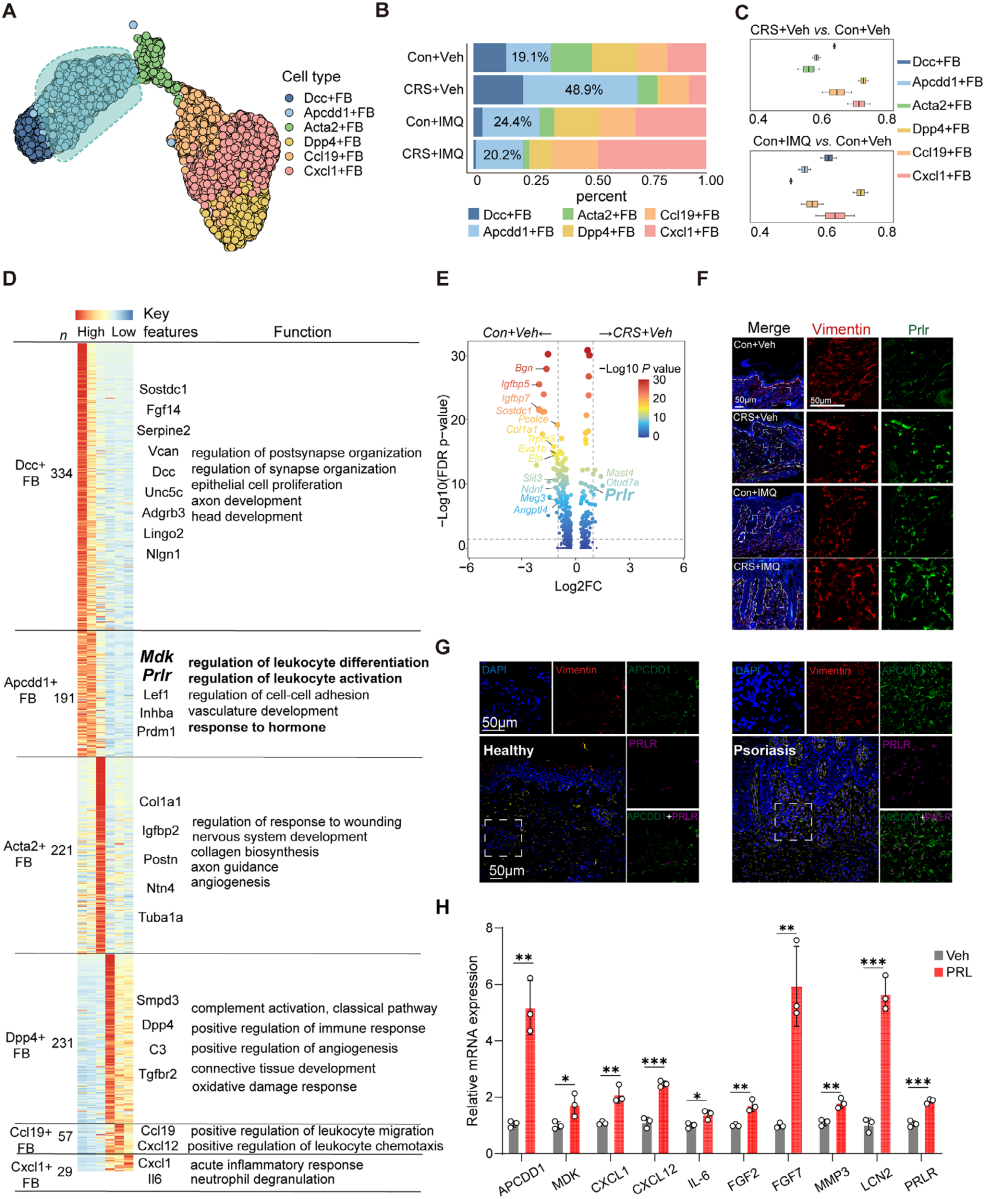
Stress influences fibroblast subcluster proportions in skin inflammation. A) UMAP of fibroblast subclusters. B) Proportion of each fibroblast subcluster across four groups. C) Purity evaluation across fibroblast subpopulations using the ROGUE algorithm. D) Heatmap of the curated gene signatures for each fibroblast cluster with the corresponding number and functional annotation listed. E) Volcano plot of DEGs in *Apcdd1* ^+^ fibroblasts in stressed vs untreated mice. F,G) Representative IF staining of PRLR expression in fibroblasts in the four mouse groups (F) and in human psoriasis vs healthy controls (G). The images presented are representative of experiments that were performed in triplicate. H) Relative gene expression of psoriasis‐associated inflammatory profiles in primary human fibroblasts treated with PRL vs Veh (*n* = 3). All results are shown as the means ± SD. ^*^ *p* < 0.05, ^**^ *p* < 0.01, ^***^ *p* < 0.001, ns, not significant (H: unpaired Student's *t*‐test). Scale bar, 50 µm. Con: control; CRS: chronic restraint stress; FB: fibroblast; FC: fold change; IMQ: imiquimod; MDK: midkine; PRLR: prolactin receptor; UMAP: uniform manifold approximation and projection; Veh: vehicle.

Next, we aimed to clarify how chronic stress influences the fate and functional diversity of fibroblasts in skin inflammation. Six distinct fibroblast subpopulations were identified based on gene expression: *Dcc* ^+^, *Apcdd1* ^+^, *Acta2* ^+^, *Dpp4* ^+^, *Ccl19* ^+^, and *Cxcl1* ^+^ fibroblasts (**Figure** **4**A; Table S6, Supporting Information). Stress‐challenged mice showed an increase in the frequency of *Apcdd1* ^+^ fibroblasts, which were increased by 2–3 fold compared to untreated mice, constituting ≈50% of the total fibroblast population in these mice (Figure 4B; Table S7, Supporting Information), but with lower purity compared to *Cxcl1* ^+^ fibroblasts across both stress‐challenged and psoriasiform mice (Figure 4C), reflective of the transcriptional changes in *Apcdd1* ^+^ fibroblasts. Gene Ontology enrichment analysis of the top DEGs for each fibroblast subtype highlighted their unique functions across the four groups (Figure 4D; Table S8, Supporting Information), with *Apcdd1* ^+^ fibroblasts enriched for genes involved in leukocyte activation, vascular development, and hormonal responses (Figure 4D; Table S8, Supporting Information). Other fibroblast subsets, for instance, *Dcc* ^+^ fibroblasts were primarily involved in synapse organization, epithelial cell proliferation and axon development; *Acta2* ^+^ fibroblasts showed significant involvement in wound healing, collagen biosynthesis, and axon guidance; *Dpp4* ^+^ fibroblasts expressed genes associated with connective tissue development, angiogenesis, complement activation, and oxidative stress; *Ccl19* ^+^ and *Cxcl1* ^+^ fibroblasts were notable for their roles in chemotaxis, leukocyte activation, neutrophil degranulation, and acute inflammatory responses (Figure 4D; Table S8, Supporting Information). These findings highlight *Apcdd1* ^+^ fibroblasts responsiveness to hormonal signals, providing a link between stress and skin inflammation.

To explore the mechanisms by which chronic stress influences the dysfunctional identity of *Apcdd1* ^+^ fibroblasts, we compared DEGs in *Apcdd1* ^+^ fibroblasts from stress‐challenged and unstressed mice. Notably, the prolactin receptor (*Prlr*) was significantly upregulated in *Apcdd1* ^+^ fibroblasts under chronic stress (Figure 4E; Table S9, Supporting Information). Among all skin cell types, *Apcdd1* ^+^ fibroblasts exhibited the highest *Prlr* expression, surpassing both keratinocytes and immune cells (Figure S4A, Supporting Information), identifying them as primary targets of stress‐induced hormonal signaling. Although the relative proportion of *Apcdd1* ^+^ fibroblasts within the fibroblast pool declined in stress‐aggravated skin inflammation (Figure 4B), this decrease was likely due to the robust expansion of *Cxcl1* ^+^ fibroblasts. IF staining confirmed that the absolute number of *Apcdd1* ^+^ fibroblasts increased in CRS+IMQ mice compared with Con+IMQ controls (Figure 4F; Figure S4B, Supporting Information). Thus, despite relative dilution, *Apcdd1* ^+^ fibroblasts expanded quantitatively, underscoring their pathogenic relevance in stress‐exacerbated inflammation. Additionally, IF staining confirmed that PRLR was overexpressed in *Apcdd1* ^+^ fibroblasts in lesional skin of stress‐challenged mice, regardless of IMQ treatment (Figure 4F; Figure S4B, Supporting Information). Furthermore, confocal microscopy analyses of human psoriatic skin showed increased PRLR expression in inflamed skin compared with healthy controls (Figure 4G; Figure S4C, Supporting Information). Consistent with the above data showing elevated prolactin in stressed psoriasis patients and stressed IMQ mice (Figures 1C and 2F), the density of tyrosine hydroxylase‐positive neurons was robustly increased in stress‐challenged mice (Figure S4D, Supporting Information), which suggested the elevated production of prolactin in lactotrophs.

To determine whether stress‐induced prolactin influenced the behavior of dermal fibroblasts, we isolated primary fibroblasts from healthy human skin and treated them with prolactin. Quantitative reverse transcription polymerase chain reaction (qRT‐PCR) showed that prolactin significantly increased the mRNA expression of *midkine (MDK)*, *APCDD1*, *CXCL1*, *CXCL12, IL‐6, FGF2, FGF7, MMP3*, as well as *PRLR* in primary fibroblasts (Figure 4H; Figure S4E, Supporting Information), which was validated by ELISA assays (Figure S4F, Supporting Information). These findings suggest that PRLR‐mediated activation of fibroblasts plays a role in stress‐exacerbation of skin inflammation.

### 2.5 *Apcdd1* ^+^Fibroblasts‐Derived MDK Promotes Stress‐Induced Skin Inflammation

**Figure 5:**
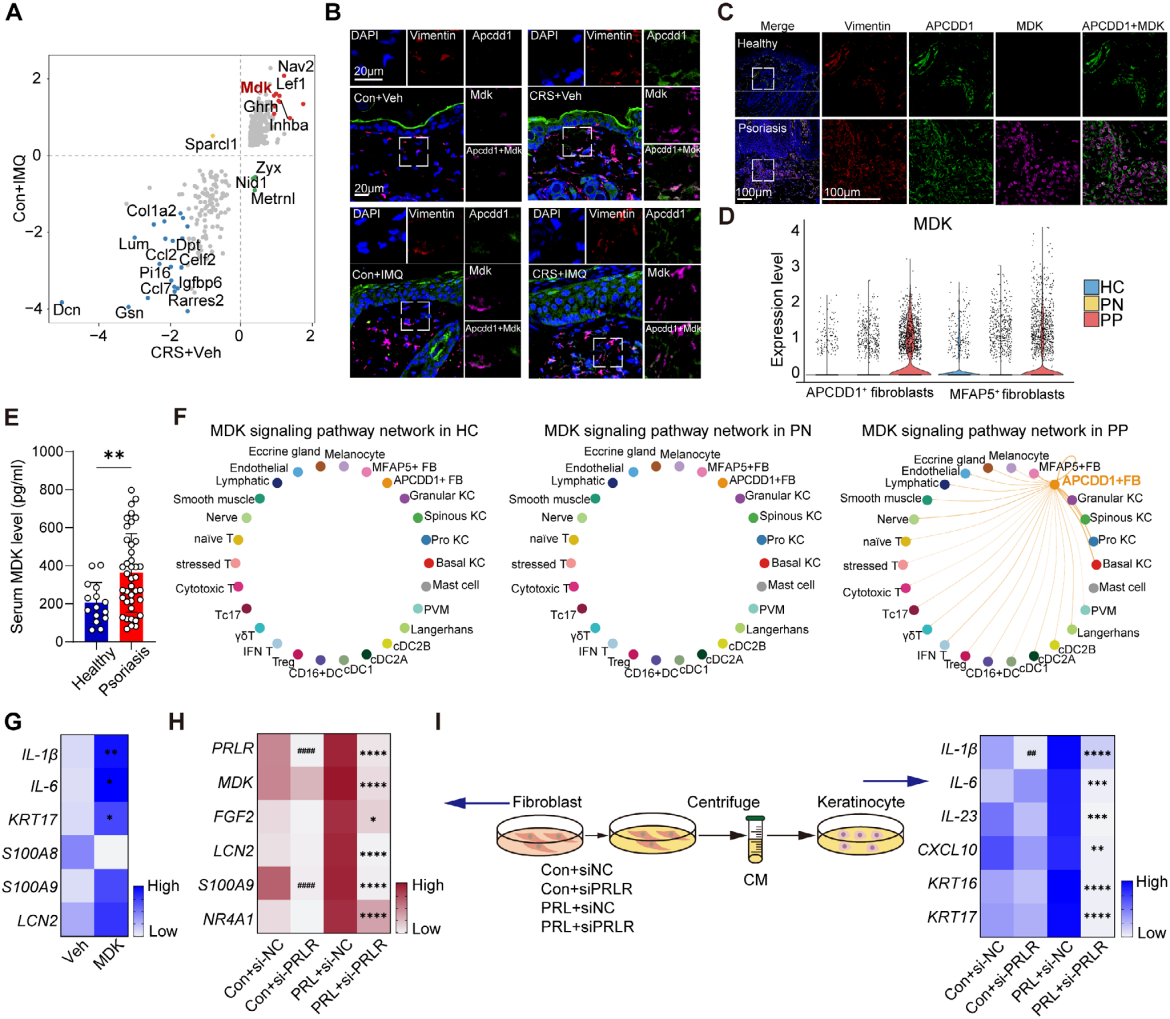
Stress‐reprogrammed fibroblasts contribute to skin inflammation via MDK. A) Overlap of DEGs identified between *Apcdd1* ^+^ vs *Apcdd1* ^−^ fibroblasts in stressed or IMQ‐challenged mice. B) Representative immunofluorescent labeling of Mdk in *Apcdd1* ^+^ fibroblasts from distinct murine skin conditions. Scale bar, 20 µm. C) Representative immunofluorescent labeling of MDK in *APCDD1* ^+^ fibroblasts from psoriasis patients vs healthy controls. Scale bar, 100 µm. D) MDK expression in fibroblasts from healthy controls, non‐lesional, and lesional areas of psoriasis. E) Plasma levels of MDK in healthy controls (*n* = 15) vs psoriasis patients (*n* = 40). F) MDK‐mediated cell‐cell communication networks from *APCDD1* ^+^ papillary fibroblasts to other cell types among healthy controls, non‐lesional, and lesional psoriasis skin. The width of the edges is proportional to the inferred strength of the interaction. G) Relative gene expression of psoriasis‐associated inflammatory profiles in primary human keratinocytes treated with MDK (*n* = 3). H) Relative gene expression of psoriasis‐associated inflammatory profiles in fibroblasts upon siPRLR transfection (*n* = 3). I) Relative gene expression of psoriasis‐associated inflammatory profiles in keratinocytes treated with fibroblast supernatant after PRL stimuli (*n* = 3). The hashtag indicates the *P* value for keratinocytes treated with conditioned medium from fibroblasts with and without siPRLR transfection before PRL stimulation, while the asterisk represents the *P* value after PRL stimulation. All results are shown as the means±SD. ^*^ *p* < 0.05, ^**/##^ *p* < 0.01, ^***^ *p* < 0.001, ^****/####^ *p* < 0.0001, ns, not significant (E and G: unpaired Student's *t* test, H and I: two‐way ANOVA). CM: conditioned medium; Con: control; CRS: chronic restraint stress; DC: dendritic cell; FB: fibroblast; HC: healthy controls; IMQ: imiquimod; KC: keratinocyte; MDK: midkine; PN: psoriatic non‐lesional skin; PP: psoriasis skin; PRLR: prolactin receptor; Pro: proliferating; PVM: perivascular macrophages; Tc17: IL17‐secreting CD8 T cells; Veh: vehicle.

We next analyzed DEGs in *Apcdd1^+^* fibroblasts, which had high expression of MDK, a secreted growth factor, under chronic mental stress and IMQ‐induced chronic dermatitis (**Figure** **5**A; Figure S5A and Table S10, Supporting Information). MDK is critical for activation of T cells and neutrophils, as well as the formation of neutrophil extracellular traps during inflammatory processes.^[^ ^22^, ^23^, ^24^ ^]^ Tissue IF co‐staining confirmed that MDK expression was increased in the *Apcdd1* ^+^ fibroblasts of stress‐challenged IMQ mice compared to non‐stressed controls (Figure 5B; Figure S5B, Supporting Information) and increased in the *APCDD1* ^+^ dermis of inflamed psoriasis skin (Figure 5C; Figure S5C, Supporting Information).To further evaluate the cross‐species conservation of MDK expression in *APCDD1* ^+^ fibroblasts, we reanalyzed publicly available single‐cell data of psoriatic skin (GSE173706) (Figure S6A–L, Supporting Information). We stratified human dermal fibroblasts by their papillary (*APCDD1, PDPN*) and reticular (*MGP, MFAP5*) localization (Figure S6F, Supporting Information), as *APCDD1* selectively marks papillary fibroblasts.^[^ ^25^, ^26^ ^]^ Consistent with the findings from IF staining of skin, MDK expression progressively increased in fibroblasts from healthy to non‐lesional to lesional areas, with the highest expression in the *APCDD1* ^+^ papillary dermis (Figure 5D). Supporting these findings, serum MDK levels were also elevated in psoriasis patients (*n* = 55, Figure 5E). Extending beyond psoriasis, fibroblast‐derived *MDK* was similarly upregulated in several psychosomatic skin disorders, including vitiligo, scleroderma, and keloids (Figure S7A–C, Supporting Information).

To investigate its pathogenic role in psoriasis, we performed cell–cell communication analysis. MDK was primarily derived from *APCDD1* ^+^ fibroblasts in lesional areas and activated signaling pathways across multiple cell types, including basal keratinocytes, *APCDD1* ^+^ fibroblasts, IFN‐γ T cells, and nerve cells (Figure 5F). To assess the effect of MDK on keratinocytes, we stimulated human primary keratinocytes with recombinant MDK and showed that MDK enhances the mRNA expression of *IL‐1β, IL‐6, and keratin 17 (KRT17)* (Figure 5G). Furthermore, deletion of the PRLR (Figure S7D, Supporting Information) markedly reduced the induction of *MDK* in PRL‐stimulated fibroblasts in addition to reduced expression of inflammatory cytokines such as *FGF2, LCN2*, and *S100A9* (Figure 5H). Supernatants from PRL‐stimulated fibroblasts further enhanced the expression of *IL‐1β, IL‐6, IL‐23, KRT16, KRT17*, and *CXCL10* in keratinocytes, which was suppressed with siPRLR in PRL‐treated fibroblasts (Figure 5I). These findings suggest that MDK is a downstream effector cytokine in prolactin‐activated fibroblasts.

### 2.6 Prolactin Promotes Fibroblast Plasticity and Skin Inflammation Through NR4A1 Activation

**Figure 6:**
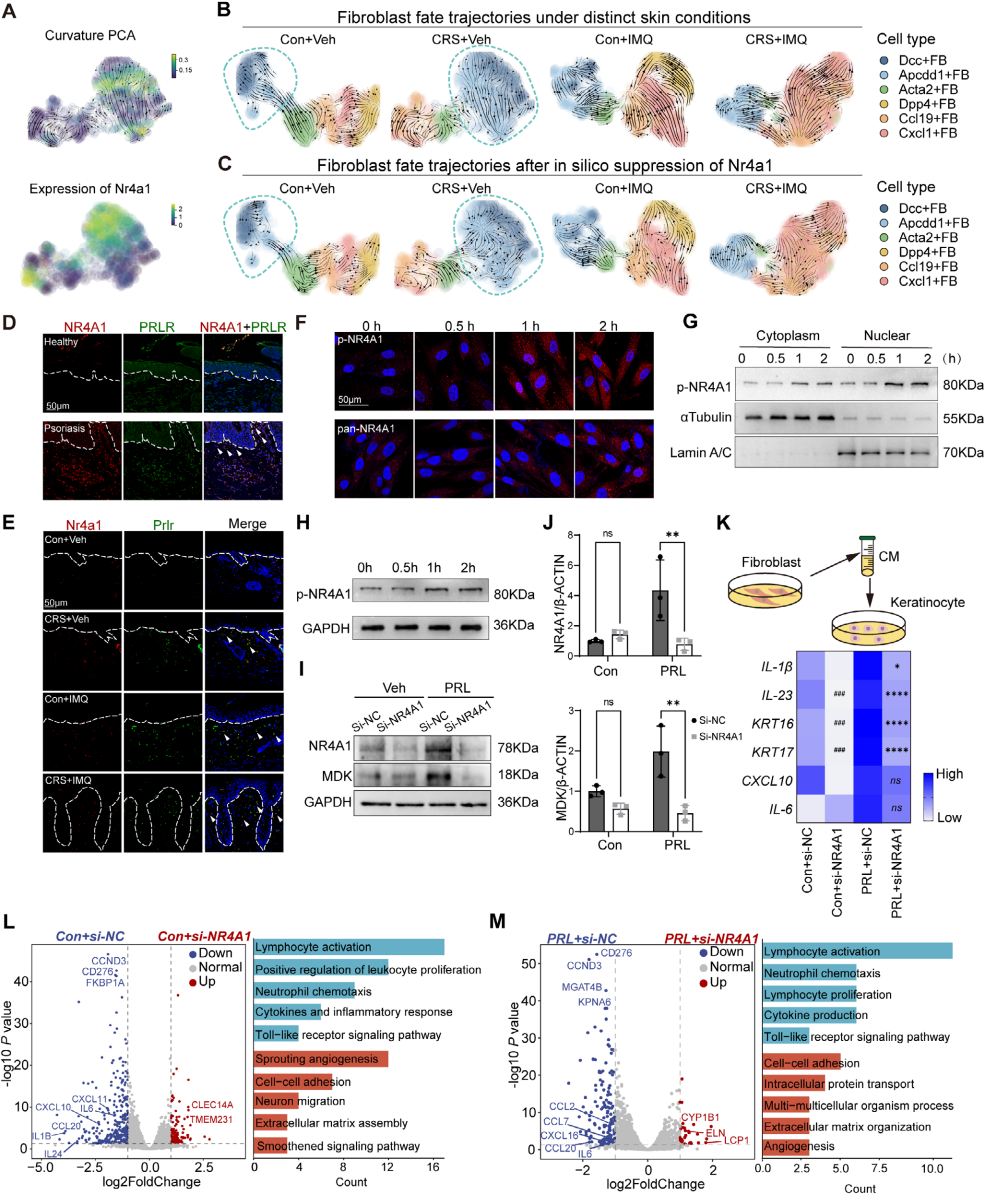
Stress modulates fibroblasts via PRL‐NR4A1 signaling. A) Cell divergence estimation (upper panel) and Nr4a1 expression (lower panel) by Dynamo. B) RNA velocity (Dynamo) estimation of fibroblast fate decision in the four study groups. C) Prediction of the fibroblast fate redirection after in silico knockout of *Nr4a1*. D) Representative immunofluorescent labeling of NR4A1 in human psoriasis vs healthy controls. E) Representative immunofluorescent labeling of Nr4a1 in the four mouse groups. F) Representative cell immunofluorescent labeling of p‐NR4A1 and pan‐NR4A1 in human fibroblasts after PRL treatment. G) Western blot of the p‐NR4A1 expression levels in nuclear and cytosolic fractions from human fibroblasts treated with PRL at different time points. H) Western blot of the p‐NR4A1 expression levels in human fibroblasts treated with PRL at different time points. I, J) Western blot analysis (I) and QRT‐PCR (J) of MDK expression after *NR4A1* knockdown in human primary fibroblasts (*n* = 3). K) Psoriasis‐related inflammatory gene expression in keratinocytes treated with conditioned medium (CM) from fibroblasts with or without siNR4A1 transfection (*n* = 3). The hashtag indicates the *P* value before PRL stimulation, and the asterisk indicates the *P* value after PRL stimulation. L,M) Volcano plots (left) and bar plots (right) showing gene expression differences and enriched pathways in fibroblasts with or without siNR4A1 transfection, before (L) and after PRL stimulation (M) (*n* = 3). All results are shown as the means ± SD. ^*^ *p* < 0.05, ^**^ *p* < 0.01, ^###^ *p* < 0.001, ^****^ *p* < 0.0001, ns, not significant (J and K: two‐way ANOVA). Scale bar, 50 µm. CM: conditioned medium; Con: Control; CRS: chronic restraint stress; IMQ: imiquimod; MDK: midkine; p‐NR4A1: phosphorylated forms of NR4A1; PRL: prolactin; Veh: vehicle.

To assess for the signaling networks that are activated under stress‐challenge, we mapped transcriptomic vector fields within fibroblast subpopulations. The expression of *Nr4a1* was increased in areas of fate decision in fibroblasts from stress‐challenged mice, particularly in the *Apcdd1^+^* fibroblast subset (**Figure** **6**A). Interestingly, the trajectory from *Apcdd1^+^* fibroblasts to *Dcc* ^+^ fibroblasts in untreated mice was completely reversed in stressed and imiquimod‐challenged mice; in contrast, the transitions from *Ccl19* ^+^ and *Cxcl1* ^+^ fibroblasts to *Dpp4* ^+^ fibroblasts remained unchanged in stressed mice (Figure 6B; Figure S8A, Supporting Information). Although there was a lower relative prevalence of *Apcdd1* ^+^ fibroblasts compared to *Cxcl1* ^+^ fibroblasts in skin inflammation (Figure 4B), it is important to highlight that other subsets of dermal fibroblasts, including *Cxcl1* ^+^ fibroblasts, were transformed into *Apcdd1* ^+^ fibroblasts (Figure S8A, Supporting Information), indicating *Apcdd1* ^+^ fibroblasts are functionally pivotal in the stress‐inflammation axis. Moreover, prediction from in silico knockout of *Nr4a1* predicted a loss of identity for *Apcdd1^+^* fibroblasts under stress challenge, promoting transition toward *Dcc* ^+^ fibroblasts and reversing the transition from *Dpp4* ^+^ fibroblasts to *Cxcl1* ^+^ fibroblasts (Figure 6C). IF revealed co‐localization of elevated NR4A1 and PRLR, specifically in *APCDD1* ^+^ fibroblasts in lesional psoriasis skin (Figure 6D), consistent with what was seen in stress‐challenged mice (Figure 6E).

To investigate the regulatory effects of prolactin on downstream effector molecules, human primary dermal fibroblasts were stimulated with prolactin and harvested at defined time points (0, 0.5, 1, and 2 h) for molecular analyses. Prolactin treatment induced a pronounced subcellular redistribution of NR4A1, with significant accumulation of phosphorylated NR4A1 in both nuclear and cytosolic fractions at 1 h post‐stimulation (Figure 6F–H; Figure S8B,C, Supporting Information). NR4A1 siRNA transfection (Figure S8D, Supporting Information) markedly suppressed IL‐6 expression in fibroblasts and abolished the induction of *Apcdd1* ^+^ fibroblasts‐derived MDK and *Cxcl1* ^+^ fibroblasts‐derived IL‐6 was nearly abolished following NR4A1 knockdown (Figure 6I,J; Figure S8E,F, Supporting Information). In addition, supernatants from PRL‐treated fibroblasts induced *KRT16, KRT17, IL‐23*, and *IL‐1β* expression in keratinocytes, dependent upon NR4A1 signaling (Figure 6K).

To further address the role of NR4A1 in PRL responses in fibroblasts, we performed RNA‐sequencing in fibroblasts with siNR4A1 transfection and compared against control (siNC) with or without PRL stimulation (*n* = 3). We identified 460 DEGs in unstimulated fibroblasts with siNR4A1, whereas 177 DEGs with PRL stimulation (FC > 2 or < 0.5; FDR < 0.05) (Figure 6L,M; Tables S11 and S12, Supporting Information). Gene Ontology analysis of NR4A1‐regulated genes in unstimulated fibroblasts showed enrichment for biological processes including lymphocyte activation (*CD38*), neutrophil chemotaxis (*CXCL2, CXCL10*), and inflammatory response (*IL1B*) (Figure 6L; and Table S13, Supporting Information). NR4A1‐regulated genes in fibroblasts under PRL stimulation were enriched for extracellular matrix organization (*CYP1B1, ELN*) and angiogenesis (*NDP, CLEC11A*) (Figure 6M; Table S14, Supporting Information).

**Figure 7:**
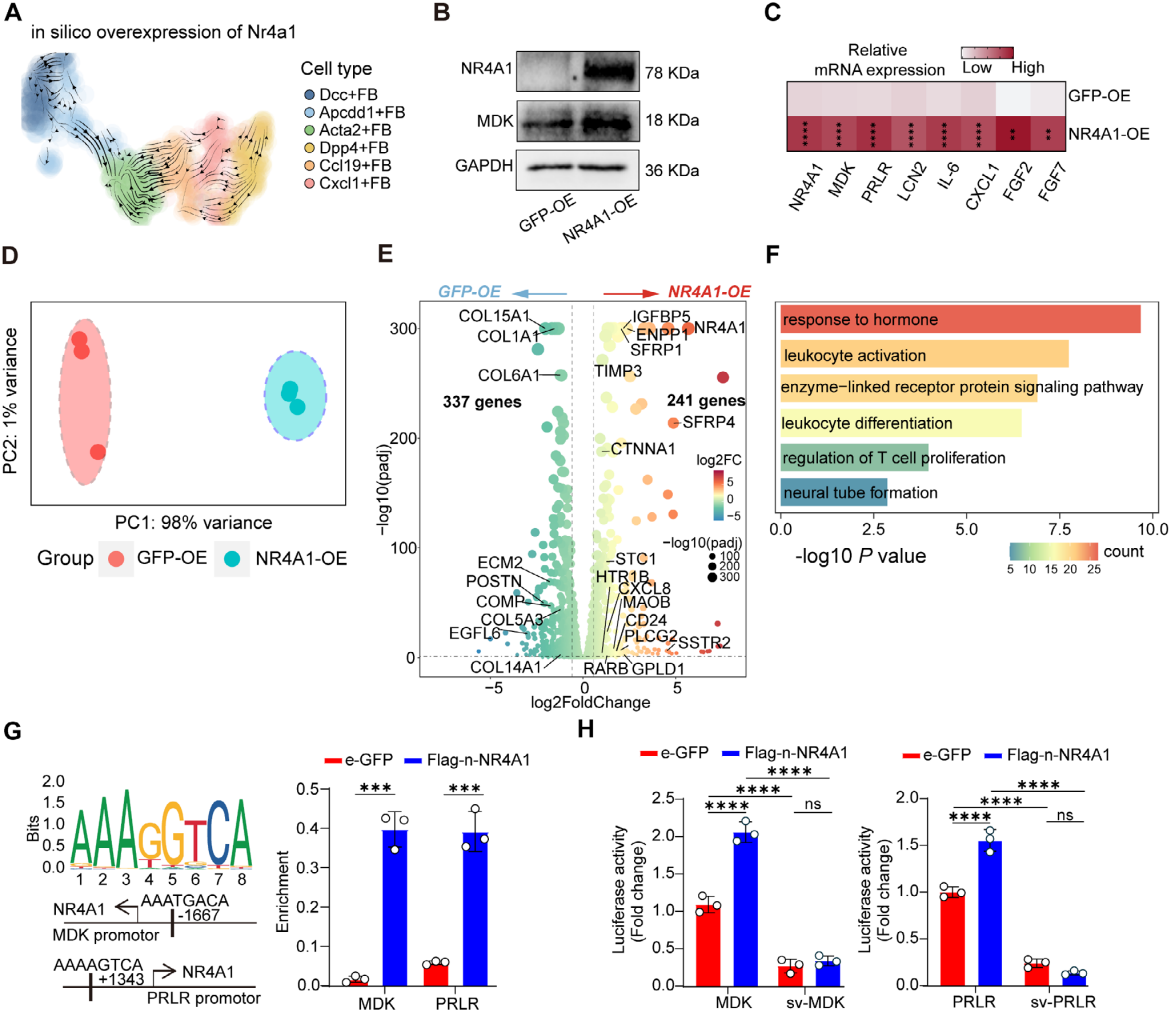
Overexpression of NR4A1 primes fibroblasts toward a hyperinflammatory phenotype. A) Prediction of the fibroblast fate redirection after in silico overexpression of Nr4a1. B) Western blot analysis of MDK expression in *NR4A1*‐overexpressing fibroblasts. C) Relative gene expression levels of inflammatory profiles in *NR4A1*‐overexpressing fibroblasts (*n* = 8–9). D) PCA data of primary human fibroblasts with and without *NR4A1* overexpression. E) Volcano plot of DEGs in fibroblasts with or without NR4A1 overexpression (*n* = 3). F) Enriched pathways in NR4A1‐overexpressing fibroblasts. Count indicates the number of genes enriched in corresponding pathways. G) Description of the NR4A1‐binding sites in the MDK (up) or PRLR (down) promoter (left panel). Primary fibroblasts transfected with lentivirus encoding e‐GFP or Flag‐n‐*NR4A1* for ChIP assay with antibodies against NR4A1 (right panel) (*n* = 3). H) Effects of NR4A1 overexpression on the wild‐type or sequence variant promoter activity of the MDK or PRLR gene in transfected fibroblasts (*n* = 3). All results are shown as the means±SD. ^**^ *p* < 0.01, ^***^ *p* < 0.001, ^****^ *p* < 0.0001, ns, not significant. (C and G: unpaired Student's *t* test; H: two‐way ANOVA). IMQ: imiquimod; MDK: midkine; OE: overexpression; PCA: principal components analysis; p‐NR4A1: phosphorylated forms of NR4A1; PRL: prolactin; sv: sequence variant; Veh: vehicle.

Modeling in silico overexpression of *Nr4a1* supported its role in mediating differentiation from *Dcc^+^* toward *Mdk*‐expressing *Apcdd1^+^* fibroblasts (**Figure** **7**A). To validate these findings, we overexpressed *NR4A1* in primary human fibroblasts and observed that they exhibited high levels of *MDK, LCN2, IL‐6, CXCL1, FGF2*, and *FGF7* (Figure 7B,C). Supernatants from NR4A1‐overexpressing fibroblasts also showed upregulation of LCN2 and IL‐6 (Figure S9A, Supporting Information). To assess NR4A1 function more broadly, we performed RNA‐seq analysis of *NR4A1*‐overexpressing fibroblasts vs GFP control (*n* = 3). Principal Component Analysis (PCA) showed a clear separation between the two groups (Figure 7D), with 241 upregulated and 337 downregulated DEGs in *NR4A1*‐OE fibroblasts (FDR < 0.05 and FC > 2 or < 0.5) (Figure 7E; Table S15, Supporting Information). Enriched biological processes included response to hormone, leukocyte activation and differentiation (Figure 7F; Table S16, Supporting Information), aligning with the findings from *Apcdd1* ^+^ fibroblasts (Figure 4D). Correspondingly, conditioned medium from *NR4A1*‐OE dermal fibroblasts significantly enhanced neutrophil migration in transwell assays (Figure S9B,C, Supporting Information). To determine whether NR4A1 directly regulated MDK or PRLR expression in fibroblasts, we analyzed the Jasper database and identified sequences in the promoter region of *MDK* and *PRLR* genes that bind to NR4A1 (Tables S17 and S18, Supporting Information). These findings were confirmed in NR4A1‐overexpressing fibroblasts using a ChIP assay, which showed that increased binding of NR4A1 to AAATGACA and AAAAGTCA sequences in the promoter regions of MDK and PRLR (Figure 7G; Tables S17 and S18, Supporting Information). To further validate these findings, we cloned the promoter region (≈2.0 kb upstream of the transcription start site for MDK or PRLR) into a luciferase reporter construct, creating a deletion that lacked the predicted NR4A1‐binding sites. The activities of the MDK and PRLR promoters were significantly increased by NR4A1 overexpression, which was abolished by the deletion of the NR4A1‐binding site (Figure 7H).

Therefore, NR4A1 functions as a core transcriptional mediator of the stress‐to‐inflammation switch.

### 2.7 Inhibiting NR4A1/MDK Signaling Suppresses Skin Inflammation In Vivo

**Figure 8:**
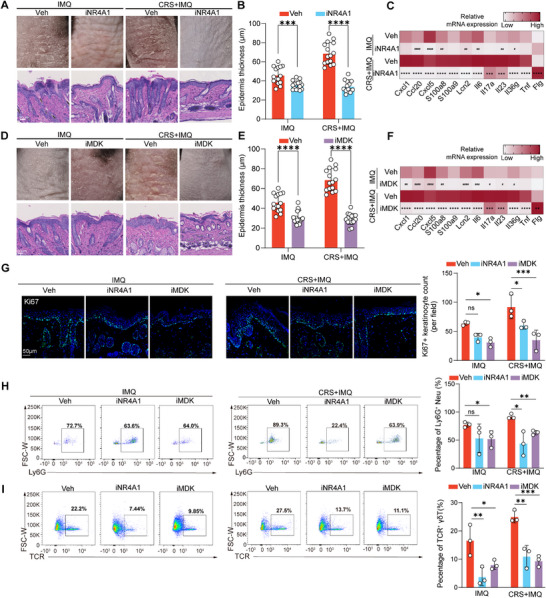
Targeting NR4A1 or MDK alleviates stress‐aggravated skin inflammation in vivo. A) Representative macroscopic views (upper panel) and H&E staining (lower panel) of IMQ‐challenged mice with and without Nr4a1 inhibition (*n* = 3). B) Quantification of epidermis thickness after Nr4a1 inhibition (*n* = 3). Each data point represents one view in corresponding groups. C) Transcriptional levels of inflammatory genes with and without Nr4a1 inhibition (*n* = 3). D) Representative macroscopic views (upper panel) and H&E staining (lower panel) with and without Mdk inhibition (*n* = 3). E) Quantification of epidermis thickness after Mdk inhibition (*n* = 3). F) Transcriptional levels of inflammatory genes with and without Mdk inhibition (*n* = 3). G) Quantitation of Ki67^+^ epidermal cells in mouse skin sections via IF. The images presented are representative of experiments performed in triplicate. (H and I) FCM analysis of peripheral neutrophils (H) and γδT cells (I) to validate the effect of Nr4a1 and Mdk inhibition (*n* = 3). All results are shown as the means ± SD. ^*/#^ *p* < 0.05, ^**/##^ *p* < 0.01, ^***/###^ *p*<0.001, ^****/####^ *p* < 0.0001, ns, not significant (The hashtag denotes the *P* value from the comparison between the IMQ‐challenged mice with and without Nr4a1/Mdk inhibition, whereas the asterisk represents the *P* value from the comparison of stressed IMQ‐challenged mice with and without Nr4a1/Mdk inhibition. B, C, E, F, G, H and I: two‐way ANOVA). Scale bar, 50 µm. CRS: chronic restraint stress; FCM: flow cytometry; IMQ: imiquimod; MDK: midkine; Veh: vehicle.

To assess the potential therapeutic impact of targeting NR4A1/MDK signaling in skin inflammation, we further intraperitoneally (*i.p*.) treated mice with NR4A1 inhibitor (iNR4A1, 20 mg Kg^−1^) and MDK inhibitor (iMDK, 20 mg Kg^−1^) every two days prior to stress‐challenge and IMQ application. Treatment with NR4A1 inhibitor suppressed erythema, acanthosis, and desquamation (**Figure** **8**A,B), which was accompanied by decreased expression of inflammatory cytokines (e.g., *Cxcl1, Cxcl5, Ccl20, Il6, Il17a, Il23, Il36g, Tnf, S100a8/9, Lcn2*) (Figure 8C) and decreased proliferation of keratinocytes, infiltration of neutrophils and γδT cells into inflamed skin (Figure 8G–I). These findings were modeled with inhibition of MDK (Figure 8D–I), supporting that NR4A1 and MDK are acting in concern and primarily in promoting fibroblast‐mediated exacerbation of inflammation.

## 3 Discussion

In this study, we systematically dissect how chronic psychological stress establishes a pro‐inflammatory predisposition in skin by initiating a defined molecular cascade. Our findings demonstrate comprehensive data showing that stress elevated prolactin that acts on skin fibroblasts, driving them toward *Apcdd1* ^+^ fibroblasts, a subset of fibroblasts located in the superficial dermis with a proinflammatory state, through activation of NR4A1 signaling and induction of MDK to promote inflammatory responses in skin. While this stress‐induced remodeling alone does not provoke overt inflammation, it creates a primed microenvironment that dramatically amplifies subsequent inflammatory responses upon inflammatory challenge. These mechanistic insights may extend to other cutaneous inflammation as well as systemic disorders modulated by psychological stress.

Stress responses involve several bodily systems, including the autonomic nervous system, neuroendocrine system, metabolism, and the immune system.^[^ ^10^ ^]^ The hypothalamic‐pituitary‐adrenal (HPA) axis plays a key role,^[^ ^27^, ^28^ ^]^ and stress mediators, such as corticosteroids, prolactin^[^ ^29^ ^]^ and catecholamines,^[^ ^30^ ^]^ directly or indirectly impact peripheral systems, exacerbating diseases like inflammatory bowel disease and atherosclerosis.^[^ ^31^, ^32^ ^]^ Our research showed that the serum cortisol level was higher in the high‐stress psoriasis group compared to the low‐stress psoriasis group. However, there was no significant difference in cortisol levels between the high‐stress psoriasis group and healthy controls. This suggests that the stress‐amplified immune responses in psoriasis might involve other neuro‐mediators. Cortisol production is influenced by various factors, including circadian rhythms, age, gender, stress, mood, diet, exercise, and sleep. In psoriasis patients, the interplay of these factors can complicate the changes in cortisol levels.^[^ ^8^, ^33^ ^]^ Strikingly, in our data, prolactin emerges as the key signal during stress,^[^ ^29^, ^34^ ^]^ promoting skin inflammation. Our findings align and explain previous clinical observations, including postpartum exacerbations of psoriasis,^[^ ^35^ ^]^ serum prolactin levels decrease after treatment^[^ ^36^ ^]^ and worsening of psoriasis in patients with prolactinomas, which also often report disease worsening with tumor progression.^[^ ^37^ ^]^ Last, treatment with bromocriptine, a dopamine agonist known to inhibit prolactin secretion, alleviates psoriasis symptoms in these cases,^[^ ^38^ ^]^ further supporting the potential of hormonal therapies for psoriasis.^[^ ^39^ ^]^ However, further large‐scale prospective cohort studies with repeated PRL measurements are needed to strengthen the robustness of the associations of prolactin with both psoriasis and depressive symptoms. Thus, our data is consistent with prolactin functioning as a mediator in the “brain‐skin axis”, promoting exacerbation of skin inflammation during periods of stress.

Prolactin is a polypeptide hormone composed of 199 amino acids, primarily produced and secreted by lactotrophs in the anterior pituitary gland. Its secretion is primarily regulated by a negative feedback loop, where prolactin stimulates the release of dopamine from hypothalamic tuberoinfundibular dopaminergic (TIDA) neurons. This dopamine then inhibits further prolactin secretion by acting on the pituitary through the median eminence and hypothalamic‐pituitary blood system.^[^ ^29^ ^]^ Recent studies, however, have shown that prolactin is also synthesized outside the pituitary, including in the skin.^[^ ^40^, ^41^ ^]^ To explore the sources of prolactin in response to stress, we examined TIDA neurons (tyrosine hydroxylase‐positive) and found their activity significantly increased in stressed IMQ mice. Quantitative analysis also demonstrated significantly increased prolactin concentrations in IMQ‐exposed mice subjected to chronic psychological stress. Prolactin levels in plasma vary throughout the day and across physiological states such as pregnancy.^[^ ^42^ ^]^ While prolactin is well‐known for its role in lactation, it also influences other biological functions, including intestinal health, where it helps maintain epithelial barrier integrity.^[^ ^43^ ^]^ Additionally, prolactin has been shown to stimulate dendritic cells (DCs), promoting the production of IL‐6 and IL‐23, which drives the differentiation of regulatory T cells (Tregs) into IL‐17^+^ Foxp3^+^ T cells.^[^ ^18^ ^]^

Our data identifies fibroblasts as the major responder to prolactin, which promotes fibroblast expansion, particularly of *APCDD1* ^+^ dermis fibroblasts, their activation and plasticity, leading to the secretion of immune‐active agents that may propagate the disease state. The apparent reduction in the proportion of *Apcdd1*⁺ fibroblasts reflected the dramatic expansion of *Cxcl1^+^* fibroblasts, as confirmed by IF staining showing their numerical accumulation in inflamed skin. This highlights a broader issue of compositional distortion in single‐cell datasets, where relative proportions can obscure true cellular dynamics. Similar artifacts have been reported in other inflammatory contexts, including intestinal lymphocyte redistribution in ileitis,^[^ ^44^ ^]^ langerhans cell shifts in bullous pemphigoid,^[^ ^45^ ^]^ and fibroblast subpopulation remodeling in keloids.^[^ ^46^ ^]^ Critically, our study identifies *Apcdd1*⁺ fibroblasts (rather than keratinocytes or immune cells) as a privileged cellular hub for translating stress‐derived neuroendocrine cues into immune dysregulation during skin inflammation. By producing MDK‐enriched inflammatory mediators that potently activate surrounding keratinocytes and immune subsets, these fibroblasts emerge as central effectors, with keratinocytes and immune cells functioning primarily as secondary amplifiers downstream of fibroblast activation.

Previous studies have highlighted the involvement of hair follicles and melanocytes in neuroimmune interactions.^[^ ^47^, ^48^ ^]^ However, our results suggest that, under stress, these tissues display the most differentially expressed genes (DEGs). Despite this, our data indicate that fibroblasts are the main contributors to the changes observed in imiquimod‐challenged mice or in human psoriasis.

Notably, our work here identifies MDK as an intermediate agent mediating stress‐induced exacerbation of skin inflammation. MDK is a heparin‐binding,^[^ ^49^ ^]^ secreted growth factor.^[^ ^50^ ^]^ Recently published studies have observed MDK expression in psoriatic lesions, with keratinocytes suggested as the primary source.^[^ ^51^ ^]^ Our findings demonstrate that MDK expression occurs predominantly in dermal *APCDD1* ^+^ fibroblasts through both single‐cell and tissue IF. Notably, keratinocytes lack expression of the prolactin receptor, rendering them unresponsive to prolactin stress signals. MDK has been shown to act as a multifaceted growth factor with roles in inflammation, including modulation of macrophage^[^ ^52^ ^]^ and T cell function,^[^ ^53^ ^]^ neutrophils and neutrophil extracellular traps,^[^ ^23^, ^24^ ^]^ angiogenesis,^[^ ^54^, ^55^ ^]^ and proliferation of neural progenitors and neurite growth.^[^ ^56^ ^]^ Thus, MDK, through these various roles may contribute to the inflammatory response in skin.

A key signaling mechanism identified here, linking stress and MDK expression, is the nuclear transcription factor NR4A1. The role of NR4A1 is best established in fibrosis, where it recruits a repressor complex to TGF‐β target genes, thereby mitigating the pro‐fibrotic effects of TGF‐β.^[^ ^57^ ^]^ Small‐molecule NR4A1 agonists have shown promise in inhibiting experimental fibrosis in skin, lung, liver, and kidney models.^[^ ^57^ ^]^ NR4A1 has further demonstrated various roles in modulating inflammatory responses,^[^ ^58^, ^59^ ^]^ promotion of apoptosis,^[^ ^60^ ^]^ and T‐cell responsiveness,^[^ ^61^ ^]^ indicating a complex role in homeostasis and disease promotion. NR4A1 has also been implicated as a mediator in response to diverse hormonal cues including corticotropin‐releasing hormone.^[^ ^62^, ^63^ ^]^ This previous work and the data provided in our manuscript highlight this axis as a novel and therapeutically targetable mechanism in controlling stress‐induced skin inflammation.

This study has several limitations. These include that different mouse strains may exhibit variable responses to stress modeling.^[^ ^64^ ^]^ However, C57BL/6 mice are well‐established for modeling skin inflammation^[^ ^65^ ^]^ and were the reason for selecting this strain for these experiments. Further, to better capture the impact of stress on diverse immune cells, we recognize the need to expand our sample size for single‐cell data and incorporate a broader range of stress challenges and stress models. Last, while mouse models may never fully recapture the complexity of human disease processes, we have taken extensive measures to validate our findings using human psoriasis single‐cell data and skin samples.

In summary, this study elucidates novel mechanisms by which stress activates *APCDD1* ^+^ dermis fibroblasts to promote the progression of skin inflammation, identifying prolactin, its downstream NR4A1, and MDK as targets for alleviating stress‐exacerbated inflammation.

## 4 Experimental Section

### Sex as a Biological Variable

Sex was not considered as a biological variable due to insufficient statistical power to analyze sex‐stratified effects.

### Human Participants

Patients diagnosed with psoriasis were enrolled in this study and disease activity was scored by the PASI. All skin lesions/tissues and the surrounding 5 cm^2^ area were not treated with any therapeutic measures for at least 2 weeks before biopsy. Two dermatologists independently determined that the dissected skin tissues were representative of their disease type. Ten healthy donors undergoing plastic surgery donated surgical skin discards for tissue IF. Human blood samples were collected from outpatients, inpatients and were eligible to participate if they had plaque psoriasis for the first time; without other autoimmune or systemic diseases and were not receiving systemic treatment or biologics for at least one month before blood sample collection. Controls were collected from sex‐, BMI‐ and age‐matched healthy volunteers. A total of 450 participants (211 psoriasis and 239 controls) were selected based on the inclusion and exclusion criteria. And the median score of Patient Health Questionnaire‐9 (PHQ‐9) was taken as the threshold value to divide the patients into low mental stress group and high mental stress group. Demographic information for all patients was described in Tables S1 and S2 (Supporting Information).

### Mice

C57BL/6 J mice were purchased from Department of Laboratory Animal Medicine of the Fourth Military Medical University (Xian, Shaanxi, China). Animals were maintained in pathogen‐free condition at a constant temperature and air humidity, under a standard 12‐h light‐dark paradigm, with free access to food and water. Mice were 6–8 weeks of age at the beginning of experiments and were randomly assigned to groups of 5 mice. Age‐matched littermates were used in all experiments. All experimental procedures were approved by the Review Committee for the Use of Animals of the Fourth Military Medical University and were performed in accordance with the National Institutes of Health Guide for the Care and Use of Laboratory Animals.

### Induction of Skin Inflammation

To induce skin inflammation, the imiquimod (IMQ)‐induced psoriasis‐like mouse model was used. Mice were treated daily with topical applications of 62.5 mg IMQ cream (5% IMQ, INova Pharmaceuticals, 3 m Health Care) on the shaved dorsal surfaces for consecutive 5 days. The epidermal thickness was measured in a blinded way after completion of the 5 days of IMQ treatment.

### Restraint Stress and Treatments

To induce restraint stress, mice were placed head‐first into 50 mL polypropylene conical tubes for a 6‐h duration each day over a span of 28 consecutive days. About 20 small holes with a diameter of ≈2 mm were drilled in advance on the tube cap to prevent suffocation of mice. During non‐stress periods, mice were housed in normal cages and allowed free access to food and water.

To explore the effect of antidepressants on stressed‐induced dermatitis, stressed IMQ mice were treated with agomelatine. From the next day after the last restraint session, agomelatine was administered by daily *i.p*. injection (10 mg kg^−1^ day^−1^, Sigma) for up to 4 weeks. Control groups were administered with saline.

To investigate the fibroblast‐mediated exacerbation of skin inflammation in vivo, 20 mg kg^−1^ of DIM‐C‐pPhCO2Me, a selective NR4A1 antagonist and 20 mg kg^−1^ of MDK inhibitor (iMDK) was administered intraperitoneally every two days to inhibit functionalities of NR4A1 and MDK. DIM‐C‐pPhCO2Me and iMDK were dissolved in 100 µL of a solution containing 5% DMSO and 95% SBE‐β‐CD using ultrasound assistance. Control groups were administered with 100 µL of a solution containing 5% DMSO and 95% SBE‐β‐CD using ultrasound assistance.

### Experimental Stress Paradigm

6–8 weeks mice underwent 6 h of restraint stress for 21 consecutive days, after which they were assessed with behavior tests. Afterward, mice were shaved back hair for skin preparation, with a 2‐day recovery period before commencing a 5‐day treatment of daily IMQ applications, while the daily restraint stress was maintained. During the experiment, the weight of the mice was monitored daily to assess disease progression.

### Behavior Tests

All behavior tests were conducted within a dedicated sound‐proof behavior facility. The mice were habituated in the experimental room for 30 min before the experiment, allowing them to acclimatize to the environment. The instruments were sanitized with 75% ethanol between tests to remove any residual odors and urine. All assessments were carried out by different experimenters in a blinded fashion.

### Open Field Test (OFT)

Mice were positioned in the center of a cubic space that measured 50 cm (width) × 50 cm (length) × 45 cm (height) with access to move freely. Mouse behaviors were monitored for 15 min using an automated analysis system. The total distance, the distance traveled in the center area, and the percentage of time spent in the central area were quantified by Open Field Test Video Analysis System (Shanghai Mobile Datum Information Technology, Shanghai, China).

### Elevated Plus Maze (EPM)

The elevated plus maze was constructed with two opposing arms (OA, 30 × 5 cm), two closed arms (CA, 30 × 5 × 25 cm), and a central area (5 × 5 cm). The plus‐shaped platform was raised to a height of 50 cm from the floor. For the trial, mice were placed in the center zone facing the open arm, and a 5‐min monitoring of behavior was recorded. The time and entry numbers in the OA were calculated and analyzed with Elevated Plus Maze Video Analysis System (Shanghai Mobile Datum Information Technology, Shanghai, China).

### Histology and Inflammatory Scores

For histology, tissue samples were preserved in 4% paraformaldehyde and then embedded in paraffin. The 4 µm sections were subjected to hematoxylin and eosin (H&E) staining. The stained slides were converted into digital format using a slide scanner (HAMAMATSU Photonics, Iwata City, Japan) and subsequently analyzed with NDP2 viewer software (HAMAMATSU Photonics). The epidermal thickness was quantified, with at least five measurements taken per section to calculate an average value.

### Immunofluorescence Staining

Tissue samples were processed by removing paraffin from 4 µm sections and rehydrating them. For cultured cells (human primary fibroblasts) on coverslips, fixation was performed with 4% paraformaldehyde for 15 min, followed by permeabilization with 0.2% Triton X‐100 (93443, Sigma–Aldrich) for 10 min. After incubation in goat serum for 1 h at room temperature, the skin sections or the cells were incubated overnight at 4 °C with corresponding primary antibodies against APCDD1 (1:100, Proteintech), p‐NR4A1 (1:200, Cell Signaling Technology), NR4A1 (1:100, Proteintech), PRLR (1:100, Abcam), MDK (1:100, Santa Cruz Biotechnology), Vimentin (1:150, Abcam), Vimentin (1:100, R&D Systems), Ki67 (1:1000, Abcam), MPO (1:100, ABclonal), CD3 (1:200, Abcam), Rabbit anti‐mouse/human TH (1: 250, Proteintech) and followed by washing and incubation with secondary antibodies. Slides were mounted with DAPI (Sigma–Aldrich) stain to visualize cell nuclei and images were acquired using a confocal microscope (LSM880, Carl Zeiss). All chemical reagents and antibodies used for experiment are listed in Table S19 (Supporting Information).

### RNA Extraction and qRT‐PCR Analysis

RNA was isolated from cell or mouse tissue samples with TRIzol reagent (15596018CN, Invitrogen), and the RNA concentration and purity were determined using a spectrophotometer (N12391, Thermo Fisher Scientific, Inc.). RNA was subjected to reverse transcription into cDNA with the PrimeScript RT‐PCR Kit (Takara, Seoul, Korea). Subsequent qRT‐PCR was carried out with SYBR Green Master Mix (RR820A, TaKaRa, Japan). Gene expression levels were analyzed using the 2^–ΔΔCt^ method, with β‐actin as the internal control for normalization. The primer sequences are provided in Tables S20–S23 (Supporting Information).

### Flow Cytometry Analysis of Mouse Skin

Mouse dorsal skin specimens were mechanically excised, cut into 1 cm × 1 cm section, and placed into an EP tube containing 1 mL of Hank's Balanced Salt Solution (HBSS, H4641, Sigma–Aldrich). The skin was then thoroughly washed by manually agitating it up and down for 15 s, repeated three times. The skin was subsequently cut into pieces smaller than 0.5 mm in a 6‐well plate on ice, using Dulbecco's modified eagle medium (DMEM, 11885‐084, Gibco) without FBS, supplemented with 1 mg mL^−1^ Collagenase Type I (MedChemExpress) and 0.2 mg mL^−1^ DNase I (BioFroxx). The mixture was incubated in a 37 °C cell culture incubator for 60 min, with gentle pipetting every 20 min to mix the cells. The resulting cell suspensions were filtered through a 40 µm cell strainer. Cells were then stained with antibodies against CD45 (1:100, Biolegend), CD3 (1:100, Biolegend), Ly6G (1:100, Biolgend), TCR (1:100, Biolegend), CD103 (1:100, Biolegend). Data analysis was performed with Flowjo v9. Background fluorescence was determined using the Fluorescence Minus One (FMO) control.

### Cell Culture

Human dermal fibroblasts were obtained from healthy individuals at Xijing hospital. The fresh skin tissue was washed in triplicate in PBS. Following the removal of adipose tissue beneath the reticular dermis and epidermis on the papillary dermis, the samples were cut into pieces with a diameter of 1–2 mm and subsequently incubated with DMEM for a duration of 2 weeks at a temperature of 37 °C. The medium was replaced every 3 days, and the culture flasks were observed for the presence of fibroblasts. All cell lines were maintained at 37 °C, 5% CO_2_ and cultured in standard medium as recommended by ATCC.

### Primary Fibroblast Culture and Stimulation

The monolayer culture technique was employed to extract primary fibroblasts. The tissue was immersed in PBS with a 2% penicillin solution for a 10‐min period to eliminate blood contaminants. Following this, the subcutaneous fat and epidermis were excised using scissors. The leftover dermis was chopped into tiny pieces and arrayed in a T25 flask, ensuring a 0.5 cm gap between each piece to preserve tissue hydration. Next, 5 mL of culture medium supplemented with 10% FBS was added to the T25 flask, which was then inverted and incubated at 37 °C with 5% CO_2_ for 3 h. Post‐incubation, the flask was carefully returned to an upright position. The culture medium, enriched with 10% FBS, was refreshed every three days, and the primary fibroblasts were monitored under a microscope for successful isolation. It typically takes 14 days for primary fibroblasts to become visible. Upon reaching the desired cell density, the fibroblasts were treated with trypsin for detachment and subculture. Cells from passages 3 to 6 were utilized for further experimental procedures. Following treatment, cells and supernatants were harvested at 24 h post‐treatment for qRT‐PCR analysis. For protein detection, Western blot and ELISA assays were conducted at 48 h post‐treatment.

### SiRNA Transfection

The specific siRNAs for PRLR and NR4A1 were purchased from Tsingke Biotechnology Co., Ltd. 1 × 10^5^ cells plated on 6‐well plates were transfected with the siRNA using oligofectamine according to manufacturer's instructions.

### NR4A1 overexpression

The lentiviral vector was acquired from Huineng Company (China), and both lentiviral expression vectors and their production were meticulously constructed in accordance with the ViraPower Lentiviral Expression Systems protocol. Primary fibroblasts were plated into a 10 cm culture dishes. Upon achieving 50–70% confluence, the media were exchanged for a complete growth medium devoid of antibiotics, which also contained lentiviral stock and 10 µg mL^−1^ of polybrene. Following a 24‐h incubation period, the supernatants were substituted with complete DMEM, and the cells were cultured for an additional 24 h. Subsequently, they were examined under a fluorescence microscope. Cells exhibiting GFP expression were collected for further experiments. Each experiment was carried out in triplicate to ensure reliability.

### Chromatin Immunoprecipitation (ChIP)

The ChIP experiment was conducted with the SimpleChIP Plus Sonication Chromatin IP Kit obtained from Cell Signaling Technology (#56383). Briefly, cells were initially fixed with 1% formaldehyde in 10 cm culture dishes for a duration of 15 min. Post‐fixation, glycine solution was used to halt the crosslinking reaction, followed by cell washing and scraping to collect cell pellets. Subsequently, the nuclear fraction was separated from the cell lysate by resuspending in both the ChIP Sonication Cell Lysis Buffer and the ChIP Sonication Nuclear Lysis Buffer. Following fragmentation with Bioruptor sonication, the chromatin was mixed with either anti‐NR4A1 antibody (2 µg per reaction, proteintech) or IgG for an incubation period of 4 h at 4 °C under rotation. ChIP‐Grade Protein G beads were then introduced to the immunoprecipitation mix and incubated for an additional 2 h at 4 °C with rotation. The crosslinks were reversed by treating with 5 m NaCl and Proteinase K for 2 h at 65 °C. The purified DNA was finally isolated using spin columns as per the manufacturer's instructions. The resulting DNA was analyzed in ChIP‐qPCR. The primer sequences employed for ChIP‐qPCR are provided in Table S20 (Supporting Information).

### Dual‐Luciferase Assay

Jasper database was utilized to predict the binding site of NR4A1 in the promoter region (−2000 to 0 bp) of MDK and PRLR genes. Subsequently, the predicted binding sites were used to design report gene vectors for MDK and PRLR promoter regions, respectively. To conduct the dual‐luciferase reporter assay, we utilized the Dual‐luciferase Reporter Assay Kit (D0010) from Solarbio Technology. Luciferase activity was assessed by calculating the ratio of the firefly luciferase signal to the Renilla luciferase signal.

### Neutrophil Migration Assays

Peripheral neutrophils were isolated following a previously described protocol.^[^ ^66^ ^]^ Chemotaxis assays were conducted using a 24‐well transwell chamber with 5 µm pores to evaluate the impact of fibroblast‐derived inflammatory molecules on neutrophil migration. Freshly isolated peripheral neutrophils were suspended in serum‐free Roswell Park Memorial Institute (RPMI)‐1640 medium at a concentration of 1.5 × 10^6^ cells mL^−1^. A total of 200 µL of this neutrophil suspension was added to the upper chamber, while 500 µl of conditioned medium derived from NR4A1‐overexpressing (NR4A1‐OE) dermal fibroblasts was placed in the lower chamber. The plates were incubated at 37 °C for 30 min to allow for migration. Following incubation, the transwell chambers were carefully dehydrated, fixed, and stained with crystal violet. Migrated neutrophils on the lower surface of the transwell membrane were visualized and quantified using crystal violet staining, and the total cell count in the lower chamber was determined using a hemocytometer.

### Enzyme‐Linked Immunosorbent Assay (ELISA)

The level of serum cortisol prolactin, thyroid stimulating hormone, estradiol, growth hormone in psoriasis patients and stress‐challenged mice, and proinflammatory profiles (e.g., IL‐6, FGF7, LCN2, MDK) from fibroblast supernatant were determined by ELISA kit according to the manufacturer's instructions. The serum specimens were collected as previously described.

### Single‐Cell RNA Seq Analysis of Murine Skin Lesions

For single‐cell RNA sequencing, paired total skin samples of control, stressed, IMQ, stressed IMQ mice (*n* = 1 for each group) were collected after 28 days of restraint stress and 5 days of IMQ application. The isolation and suspensions of total cells in skin tissues, library preparation, and alignment were performed by CapitalBio Technology using the 10X Genomics platform and Illumina NextSeq. Raw cell‐gene UMI counting matrices generated from Cell Ranger (v3.0.2) were converted into a Seurat object and analyzed using Seurat (Version 4.1.0)^[^ ^67^ ^]^ in R environment (Version 4.0.5). Quality control entailed the removal of cells with high mitochondrial abundance (> 5%), low gene detection (<50), and high count detection (>6000). Normalization was then performed with NormalizeData and highly variable genes were determined using FindVariableFeatures. Subsequently, cells were integrated by SelectIntegrationFeatures, FindIntegrationAnchors and IntegrateData functions with default parameters. For an initial estimation of cell types, cells clustered using RunPCA and RunUMAP were annotated manually through canonical cell markers, while potential doublet cells marked by several lineage markers and away from main clusters were then filtered by cluster‐level approach. Unbiased clustering was conducted using the FindNeighbors and FindClusters functions with default parameters and a resolution value of 0.5. Differential gene expression was then calculated using the Seurat function FindAllMarkers and FindMarkers functions with Wilcoxon rank sum test, and the results were adjusted for multiple testing using the Bonferroni method. The packages used for transcriptional analysis are listed in Table S24 (Supporting Information).

### Single‐Cell RNA Seq Analysis of Human Skin Lesions

All Seurat objects for publicly available single‐cell RNA seq datasets were preprocessed and integrated in the previous work with Harmony package (Version 1.0).^[^ ^68^, ^69^ ^]^ To ensure comparability, immune cells in psoriasis were clustered and annotated according to Gudjonsson JE's study.^[^ ^70^ ^]^

### Pseudotime Analysis

R package Slingshot (Version1.8.0)^[^ ^71^ ^]^ was used to construct a developmental trajectory for cell lineage, while undifferentiated keratinocytes with the expression of *Krt5* and *Krt14* were set as starting points for both steady and stressed states. To run Slingshot, Seurat object with pre‐computed UMAP embeddings were transformed into a SingleCellExperiment object and served as an input to the function slingshot (reducedDim = “UMAP”, clusterlabels = “subtypes”, start.clus = “undifferentiated keratinocytes”, extend = “n”, stretch = 0).

### RNA Velocity

To generate spliced and unspliced assays used to infer RNA velocity, the command line interface tool in “velocyto” (Version 0.6)^[^ ^72^ ^]^ was performed (https://velocyto.org/). The output loom files were imported into the “dynamo” python package (Version 1.2.0)^[^ ^73^ ^]^ for detailed analysis, and metadata of each cell subtypes for RNA velocity analyses were obtained from Seurat object. Continuous vector fields of fibroblasts fate under multiple skin conditions were constructed via dyn.vf.VectorField function and genes strongly steering the cell fates were identified through dyn.vf.curvature. To estimate the cell fate diversions following *Nr4a1* knockdown, in silico perturbations were conducted to explore the fate trajectory through dyn.pd.perturbation function. All parameters in Dynamo were set according to the default parameters.

### Assessment of the Purity of Cell Subtypes

For purity analysis of dermal fibroblasts in mouse skin tissues, Ratio of Global Unshifted Entropy (ROGUE, Version 1.0)^[^ ^74^ ^]^ was used to quantify cellular transcriptional heterogeneity of each subcluster. The higher value denotes more similar functionalities, while lower value indicates greater variability in the subcluster.

### Enrichment Analysis

Library sequence and alignment of bulk skin RNA‐seq were conducted by OmicShare company. The downstream analyses were performed by DESeq2 (Version 1.38.2)^[^ ^75^ ^]^ in R environment (Version 4.2.3). Genes with log2 fold changes exceeding 1/−1 and adjusted *P* <0.05 were considered differentially expressed. Pathway enrichment analysis of significantly differentially expressed transcripts generated by Seurat pipelines and DESeq2 was performed via Metascape.^[^ ^76^ ^]^ Functional enrichment of fibroblasts subpopulations was performed on selected DEGs based on adjusted *P* value <0.05, absolute log2 fold change >1.5 and pct.1‐pct.2 > 0.25.

### Cell–Cell Communication Analysis

For investigation of MDK‐mediated cell–cell communication in psoriasis patients, CellChat (Version 1.4.0)^[^ ^77^ ^]^ was utilized to infer the influence of fibroblast‐derived signaling on other skin cells. Normalized data from Seurat object were loaded and applied standard parameters to assess signaling between cell types according to CellChatDB.human database. Highly variable ligand‐receptor pairs were identified through identifyOverExpressedGenes and identifyOverExpressedInteractions functions. Significant interaction was visualized through scatter plot and circle plot. All parameters in CellChat were set according to the default parameters.

### Western Blot Analysis

Proteins were isolated from tissue and cultured cell samples using RIPA buffer (P0013C, Beyotime, Shanghai, China) and denatured by boiling with loading buffer for 10 min. Protein concentrations were determined using a BCA Protein Assay Kit (PA115‐02, TIANGEN, Beijing, China). The prepared samples were then separated by 10% sodium dodecyl sulfate‐polyacrylamide gel electrophoresis (SDS‐PAGE), and transferred onto a PVDF membrane. The membrane was subsequently blocked with 5% BSA in PBS and probed with primary antibodies: PRLR (1:1000, Abcam), NR4A1 (1:1000, Proteintech), p‐NR4A1 (1:1000, Cell Signaling Technology), MDK (1:1000, Santa Cruz Biotechnology). After being washed with PBST, the membranes were incubated with horseradish peroxidase‐conjugated secondary antibodies. Finally, the membranes were chemiluminescence using the chemiluminescent reagents (Millipore Corporation, Billerica, MA) and visualized with the Bio‐Rad ChemiDoc XRS+ imaging system (Bio‐Rad, Hercules, CA). Cytoplasmic and nuclear protein of cells was separated by Nuclear and Cytoplasmic Protein Extraction Kit (P0028, Beyotime) according to the manufacturer's instruction.

### Study Approval

The study was carried out in compliance with the institutional guidelines and approved by the Ethical committee of the Xijing Hospital, the Fourth Military Medical University (KY20192069‐F‐1). All animal experiments were approved by the Institutional Animal Care and Use Committee of the Fourth Military Medical University (KY20233162‐1) and conducted in accordance with NIH guidelines. All subjects provided written informed consent.

### Statistical Analysis

All data were presented as mean ± standard deviation (SD) of at least three independent experiments. Statistical analyses were performed using GraphPad Prism software (version 9.0), with specific statistical methods, sample sizes (*n*), and significance levels indicated in corresponding figure legends. Comparisons between two groups were analyzed using unpaired Student's *t* tests. For comparisons involving more than two groups, parametric ANOVA was employed. The correlation between hormone levels and clinicopathological parameters was assessed using the Pearson's correlation. *P <*0.05 was considered to indicate statistical significance (^*/#^ *p* < 0.05, ^**/##^ *p* < 0.01, ^***/###^ *p* < 0.001, ^****/####^ *p* < 0.0001, ns, not significant).

## Conflict of Interest

The authors declare no conflicts of interest.

## Author Contributions

Z.L., H.Q., and W.L. contributed equally to this work. Z.L. and S.S. conceived and designed experiments. W.L., H.Q., Z.L., J.C., and M.C. conducted the majority of experiments. H.Q. and S.S. wrote the majority of the manuscript. X.T. designed the schematic diagram. K.X., X.L., J.M., Y.B., R.D., B.L., J.H., and W.G. collected skin tissues. E.D. and Q.L. interpreted the results. S.S., G.W., and J.E.G. discussed and supervised the experiment and helped revise the manuscript.

## Supporting information



Supporting Information

Supporting Information

## Data Availability

All data associated with this study are present in the paper or the Supporting Information. All data and code to understand and assess the conclusions of this research are available in the main text and supplementary materials. Single‐cell RNA‐seq and bulk RNA‐seq data have been deposited at GEO and are publicly available in the Genome Sequence Archive (GSA) in National Genomics Data Center, Beijing Institute of Genomics (BIG), Chinese Academy of Sciences. Accession numbers are listed in the Table S25 (Supporting Information). Datasets will be shared by the lead contact upon request.
