# Supplementary material for: Stress‐Induced Activation of Prolactin‐NR4A1‐Midkine Axis Exacerbates Skin Inflammation
Source: Adv Sci (Weinh). 2025 Nov 5;13(5):e09679. doi: 10.1002/advs.202509679 (PMC12850390; doi:10.1002/advs.202509679)
Supplement: Supplementary file 1 — Supporting Information [file ADVS-13-e09679-s001.docx]

**Supplementary Information for**

**Stress-induced activation of prolactin-NR4A1-midkine axis exacerbates skin inflammation**

*Zhiguo Li, Huiyi Quan, Wanting Liu, Jiaoling Chen, Mengyang Chu, Xin Tang, Ke Xue, Xuan Liu, Jingyi Ma, Yaxing Bai, Ruina Dong, Bing Li, Junfeng Hao, Wei Guo, Qingyang Li, Erle Dang, Johann E Gudjonsson^*^, Gang Wang^*^ and Shuai Shao^*^*

1. Li, H. Quan, W. Liu, J. Chen, M. Chu, X. Tang, K. Xue, X. Liu, J. Ma, Y. Bai, R. Dong, B. Li, J. Hao, W. Guo, Q. Li, E. Dang, G. Wang, and S. Shao

Department of Dermatology, Xijing Hospital, Fourth Military Medical University, Xi’an, Shaanxi 710032, China.

Email: [shaos@](mailto:shaoshuai19900728@qq.com)fmmu.edu.cn; xjwgang@fmmu.edu.cn

JE. Gudjonsson

Department of Dermatology, University of Michigan, Ann Arbor, MI 48109, USA.

Email: johanng@med.umich.edu

Zhiguo Li, Huiyi Quan, and Wanting Liu, contributed equally to this work.

**This file includes:
Supplementary Figures and Figure legends:**

Figure S1. The level of stress-related hormones in psoriasis patients and healthy controls, related to Figure 1.

Figure S2. Chronic restraint stress induces depressive and anxiety-like behaviors in mice, related to Figure 2.

Figure S3. Stress modulates inflammatory phenotypes across diverse cell types, related to Figure 3.

Figure S4. Chronic stress contributes to the stress-induced inflammatory phenotype of fibroblasts through PRLR, related to Figure 4.

Figure S5. MDK expression in *Apcdd1*^+^ fibroblasts from stress-challenged mice and human psoriasis, related to Figure 5.

Figure S6. Identification of cell types observed across healthy, non-lesional, and lesional areas of psoriasis, related to Figure 5.

Figure S7. Elevated levels of MDK in dermal fibroblasts across several different psychosomatic skin disorders, related to Figure 5.

Figure S8. Prolactin activates immune response of dermal fibroblasts via NR4A1, related to Figure 6.

Figure S9. Overexpression of NR4A1 leads to the release of psoriasis-associated molecules in fibroblasts, related to Figure 7.

**Supplementary Tables and Table legends (details shown in attached Excel files):**

Table S1. Characteristics of the Study Population, related to Figure 1.

Table S2. Patient characteristics for serum hormone detection, related to Figure 1.

Table S3. Differentially expressed genes of cell types across several skin conditions, related to Figure 3.

Table S4. Count of various cell types in different groups, related to Figure 3.

Table S5. Differentially expressed genes of cell types in stressed mice *vs.* untreated mice, related to Figure 3.

Table S6. Differentially expressed genes of fibroblast subtypes across several skin conditions, related to Figure 4.

Table S7. Count of fibroblast subtypes in four skin conditions, related to Figure 4.

Table S8. Functionalities of fibroblast subtypes, related to Figure 4.

Table S9. Differentially expressed genes of *Apcdd1*^+^ fibroblasts in stressed mice *vs.* untreated mice, related to Figure 4.

Table S10. Differentially expressed genes of *Apcdd1*^+^ fibroblasts *vs.* *Apcdd1*^-^ fibroblasts in stressed or psoriasiform mice, related to Figure 5.

Table S11. Differentially expressed genes of fibroblasts without prolactin stimuli in siNR4A1 *vs.* siNC transfection, related to Figure 6.

Table S12. Differentially expressed genes of fibroblasts with prolactin stimuli in siNR4A1 *vs.* siNC transfection, related to Figure 6.

Table S13. Enrichment analysis of fibroblasts without prolactin stimuli in siNR4A1 *vs.* siNC transfection, related to Figure 6.

Table S14. Enrichment analysis of fibroblasts with prolactin stimuli in siNR4A1 *vs.* siNC transfection, related to Figure 6.

Table S15. Differentially expressed genes of fibroblasts with and without NR4A1 overexpression, related to Figure 7.

Table S16. Enrichment analysis of fibroblasts with NR4A1 overexpression, related to Figure 7.

Table S17. Depiction of the NR4A1‒binding site in MDK promoter, related to Figure 7.

Table S18. Depiction of the NR4A1‒binding site in PRLR promoter, related to Figure 7.

Table S19. Antibodies, samples and chemical regents.

Table S20. Chip sequence used for quantitative PCR, related to Methods.

Table S21. SiRNA primer sequences used for quantitative PCR, related to Methods.

Table S22. Primers for qPCR of human genes, related to Methods.

Table S23. Primers for qPCR of mouse genes, related to Methods.

Table S24. Software and algorithms.

Table S25. Transcriptomic datasets used in this study.

**Figures and Figure legends**


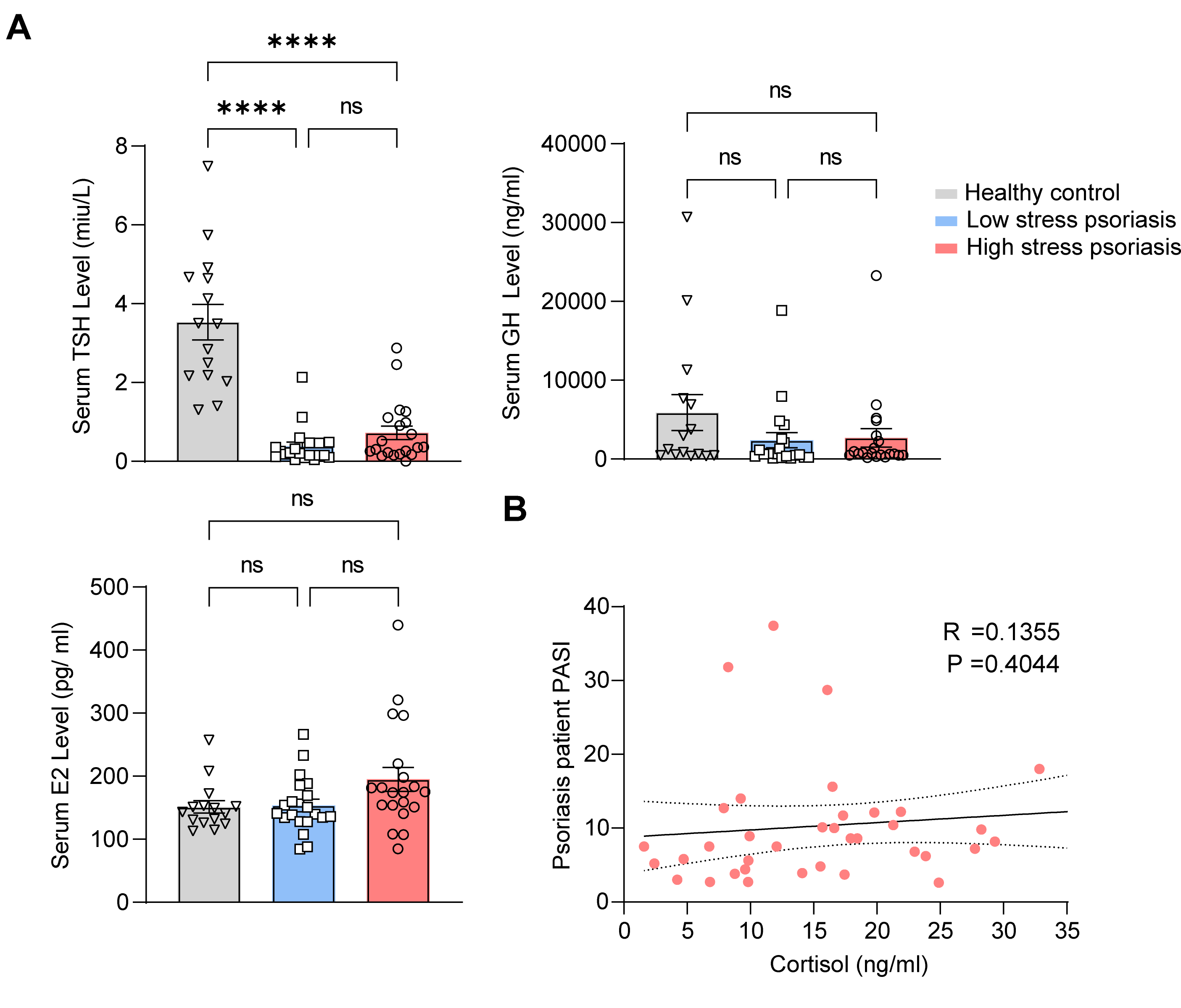


**Figure S1.** The level of stress-related hormones in psoriasis patients and healthy controls, related to Figure 1. A) Bar plot of plasma concentrations of TSH, E2, GH in control (*n* =15), unstressed (*n* = 20), and stressed psoriasis patients (*n* = 20). B) Pearson’s correlation of serum cortisol level and disease severity (PASI). All results are shown as the means±SD. ^****^*p* < 0.0001, ns, not significant (one-way ANOVA). TSH: thyroid stimulating hormone; E2: estradiol; GH: growth hormone; PASI: Psoriasis area and severity index.


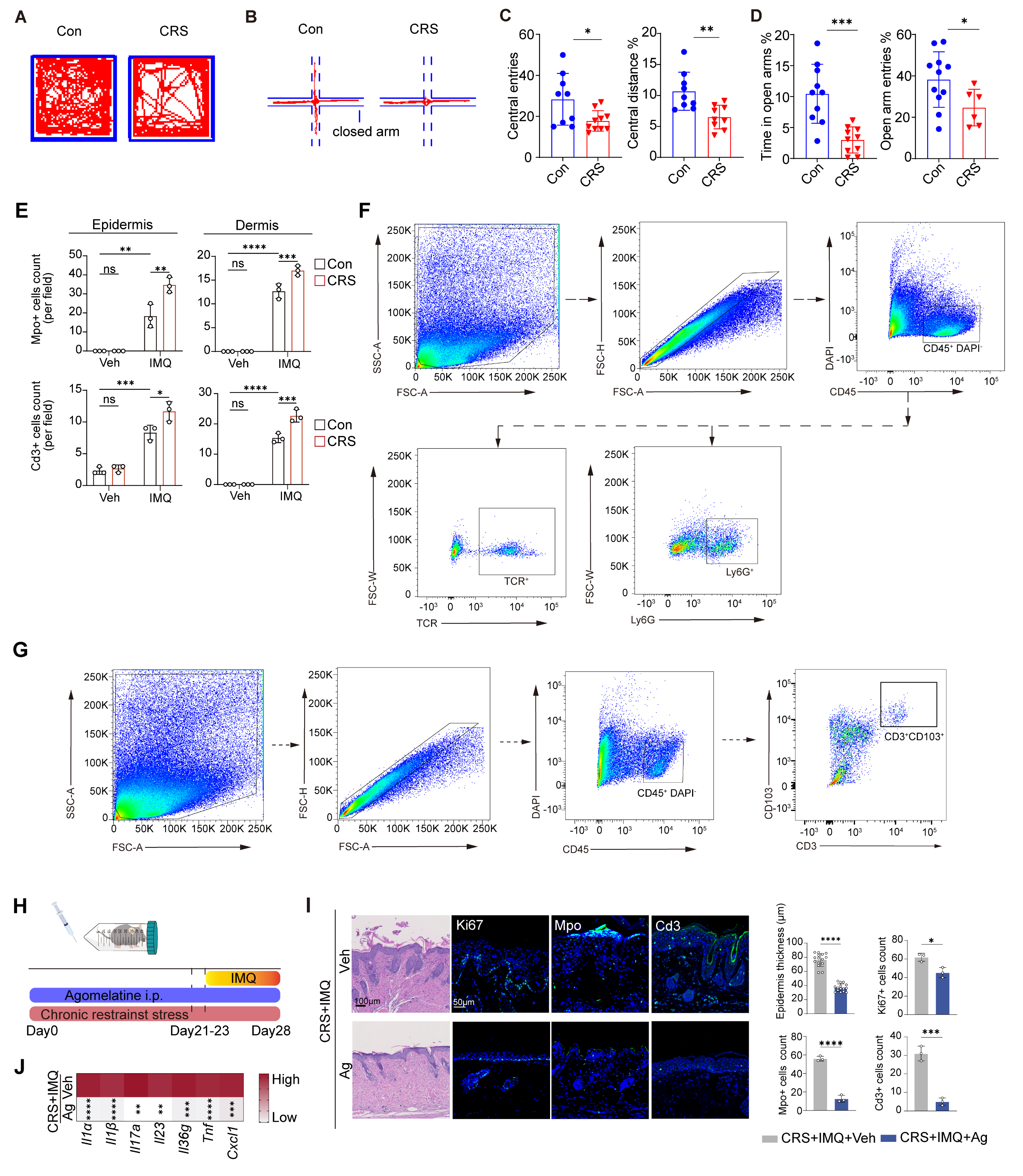


**Figure S2.** Chronic restraint stress induces depressive and anxiety-like behaviors in mice, related to Figure 2. A, B) Representative traces of mice with and without chronic mental stress in open field test (A) and in the elevated plus maze (B) (*n* = 6~11). C, D) Statistical analysis of open field test (C) and elevated plus maze (D) (*n* = 6~11). E) Quantitation of Mpo^+^ neutrophils and Cd3^+^ T cells by their location in mouse skin (*n* = 3). F, G) Gating strategies for neutrophils, γδT cells (F) and tissue resident memory T cells (G) in skin lesions. H) Schematic of the construction process of stressed psoriasiform mice with antidepressant therapy. I) Quantitation of skin thickness, Ki67^+^ epidermal cells, Mpo^+^ neutrophils and Cd3^+^ T cells of back skin from stressed psoriasiform mouse with and without Ag treatment (*n* = 3). J) Transcriptional levels of inflammatory genes in stressed psoriasiform mouse with and without Ag (*n* = 3). All results are shown as the means±SD. ^*^*p* < 0.05, ^**^*p* < 0.01, ^***^*p* < 0.001, ^****^*p* < 0.0001 (C, D, I and J: unpaired Student’s *t* test, E: two-way ANOVA). Ag: Agomelatine; Con, control; CRS: chronic restraint stress; IMQ: imiquimod; Veh: vehicle.


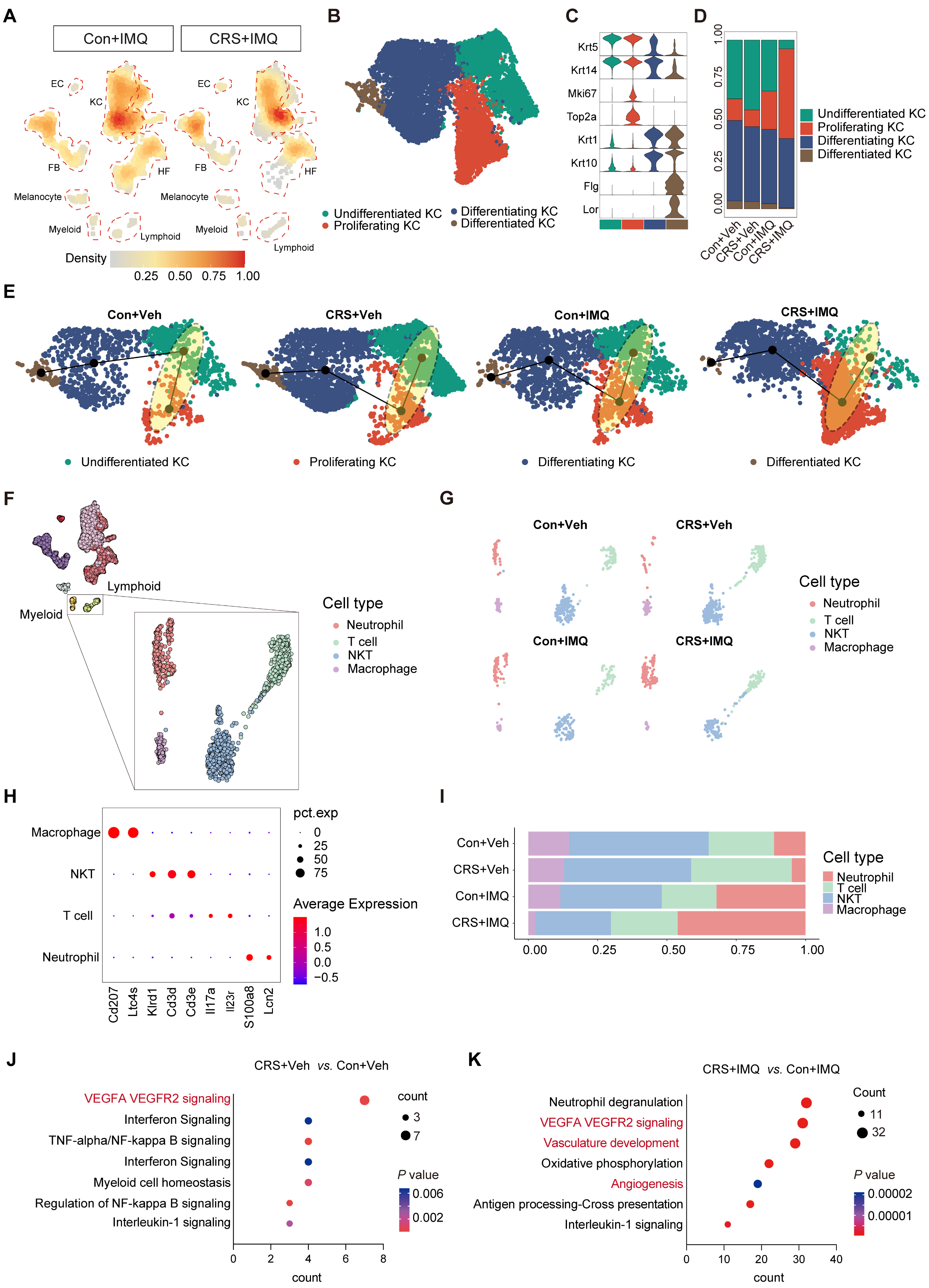


**Figure S3.** Stress modulates inflammatory phenotypes across diverse cell types, related to Figure 3. A) UMAP view of cluster density displaying skin cell distribution across distinct groups. Higher relative cell density is shown as bright orange. B) UMAP plot of keratinocytes colored by cell subtypes. C) Violin plot visualization of marker gene expression in keratinocytes. D) Relative abundance of keratinocyte subclusters in each condition. E) UMAP embedding of trajectories of keratinocytes differentiation across steady and stressed atlas with undifferentiated keratinocytes set as root. F) UMAP embedding of immune cells after subclustering of myeloid and lymphoid cells. G) UMAP embedding of the distribution of immune cells from distinct groups. H) Dot plot visualization of marker gene expression of each cell type. I) Relative abundance of myeloid and lymphoid cells in each condition. J, K) Enrichment analysis of DEGs identified in endothelial cells from CRS+Veh *vs*. Con+Veh (J) or CRS+IMQ *vs*. Con+IMQ (K). Con: control; CRS: chronic restraint stress; DEGs: differentially expressed genes; EC: endothelial cell; FB: fibroblast; HF: hair follicle; IMQ: imiquimod; KC: keratinocyte; NKT: natural killer T cell; UMAP: uniform manifold approximation and projection; Veh: vehicle.


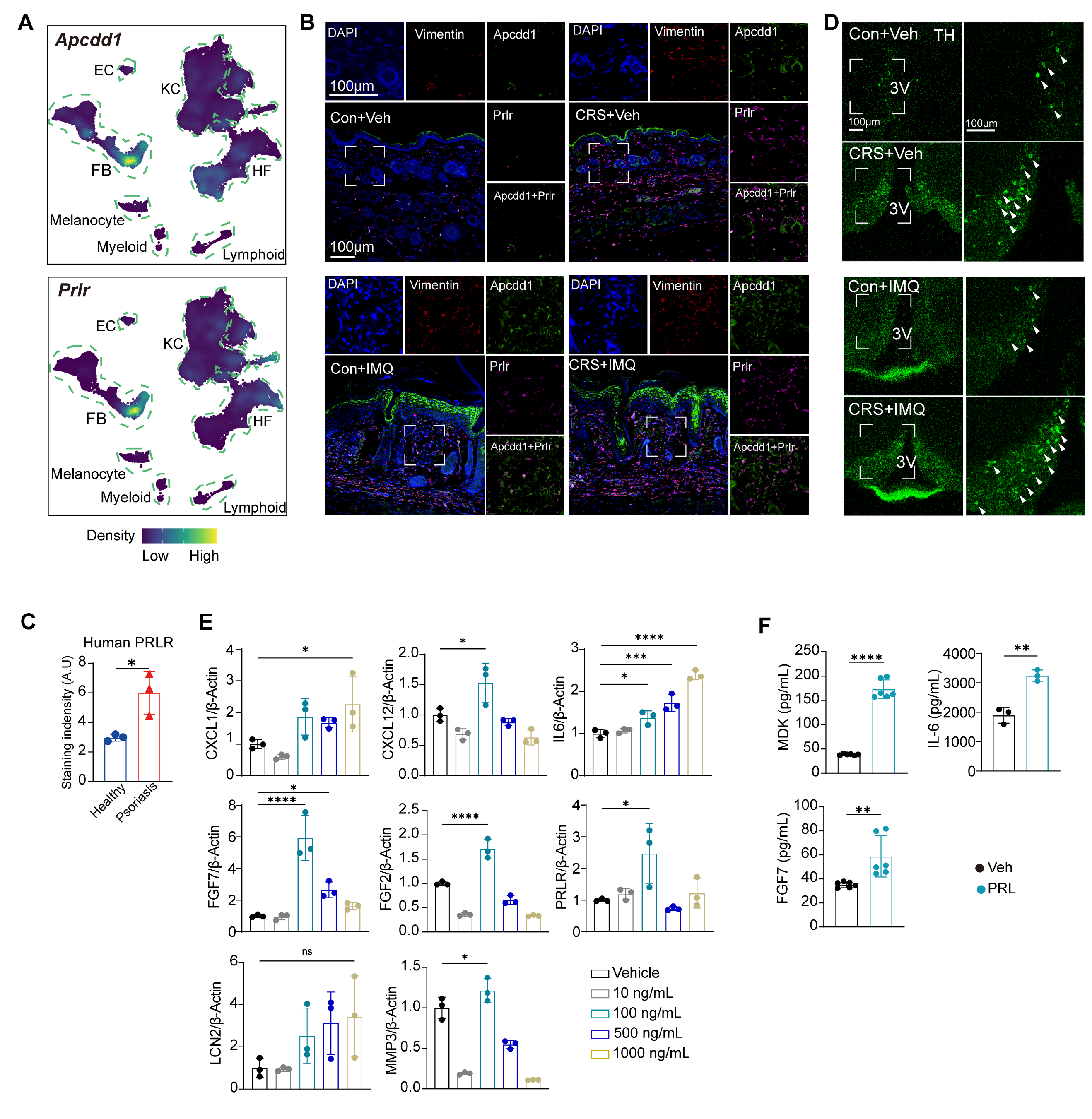


**Figure S4.** Chronic stress contributes to the stress-induced inflammatory phenotype of fibroblasts through PRLR, related to Figure 4. A) UMAP visualization of *Apcdd1* and *Prlr* expression in several cell types. B) Representative immunofluorescent labeling of *Prlr* in *Apcdd1*^+^ fibroblasts from distinct murine skin conditions. Scale bar, 100 μm. C) Evaluation of mean fluorescence intensity of PRLR expression in human psoriasis *vs*. healthy controls (*n* = 3). D) Representative immunofluorescent staining of tyrosine hydroxylase-positive neurons in the four mouse groups. Scale bar, 100 μm. E) Relative expression of proinflammatory profiles in fibroblasts treated with varying concentrations of prolactin (*n* = 3). F) Detection of inflammatory molecules by ELISA using conditioned medium collected from fibroblasts with and without PRL stimuli (*n* = 3~6). All results are shown as the means±SD. ^*^*p* < 0.05, ^**^*p* < 0.01, ^***^*p* < 0.001, ^****^*p* < 0.0001, ns, not significant (C, F: unpaired Student’s *t* test, E: one-way ANOVA). A.U: Arbitrary Unit; Con: control; CRS: chronic restraint stress; EC: endothelial cell; FB: fibroblast; HF: hair follicle; 3V: third ventricle; IMQ: imiquimod; KC: keratinocyte; PRL: prolactin; Veh: vehicle.


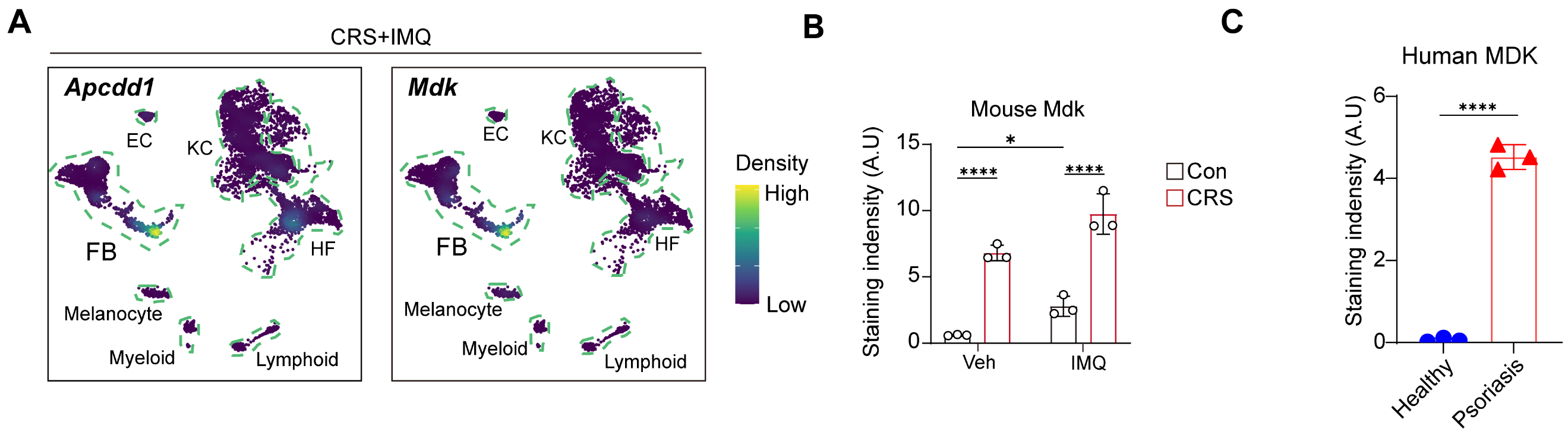


**Figure S5.** *MDK* expression in *Apcdd1*^+^ fibroblasts from stress-challenged mice and human psoriasis, related to Figure 5. A) UMAP visualization of *Apcdd1* and *Mdk* expression in several cell types from CRS+IMQ group. B) Evaluation of mean fluorescence intensity of Mdk expression in the four mouse groups (*n* = 3). C) Evaluation of mean fluorescence intensity of MDK expression in human psoriasis vs. healthy controls (*n* = 3). All results are shown as the means±SD. ^*^*p* < 0.05, ^****^*p* < 0.0001 (B: two-way ANOVA, C: unpaired Student’s *t* test). A.U: Arbitrary Unit; Con: control; CRS: chronic restraint stress; EC: endothelial cell; FB: fibroblast; HF: hair follicle; IMQ: imiquimod; KC: keratinocyte; Mdk: midkine; Veh: vehicle.


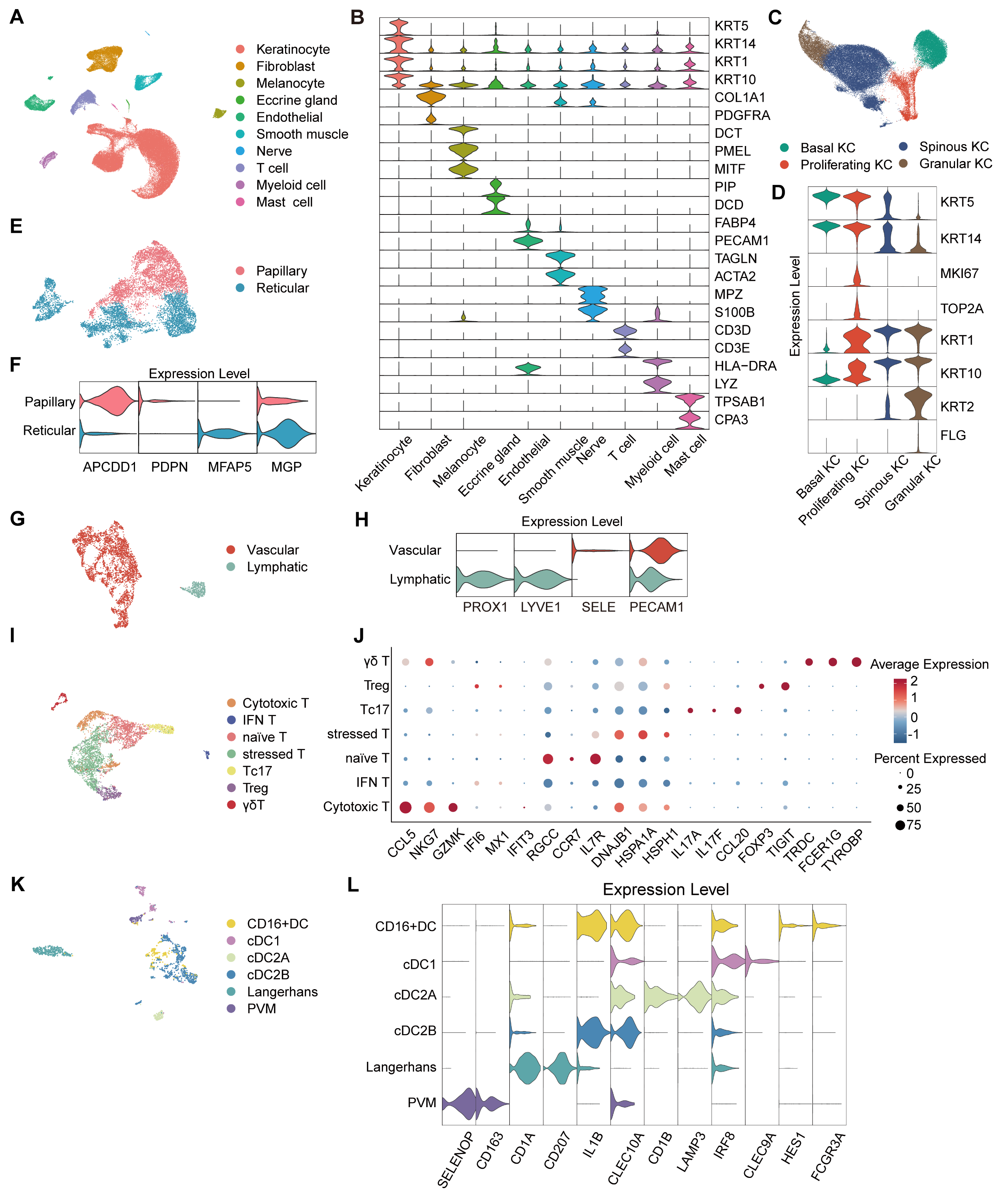


**Figure S6.** Identification of cell types observed across healthy, non-lesional, and lesional areas of psoriasis, related to Figure 5. A) UMAP visualization of skin cells colored by corresponding cell types (GSE173706). B) Violin plot of representative marker genes expression for each cell type. C) UMAP plot of keratinocytes colored by subtypes. D) Violin plot of representative marker genes expression for keratinocytes subclusters. E) UMAP visualization of dermal fibroblasts colored by subtypes. F) Violin plot of representative marker genes expression for fibroblasts subclusters. G) UMAP visualization of endothelial cells colored by subtypes. H) Violin plot of representative marker genes expression for endothelial subclusters. I) UMAP visualization of T cells colored by subtypes. J) Dot plot of representative marker genes expression for each subcluster in T cells. K) UMAP visualization of myeloid cells colored by subtypes. L) Violin plot of representative marker genes expression for myeloid subclusters. cDC: conventional dendritic cell; IFN: interferon; KC: keratinocyte; PVM: perivascular macrophage; Tc17: IL17-secreting CD8^+^T cell; UMAP: uniform manifold approximation and projection.


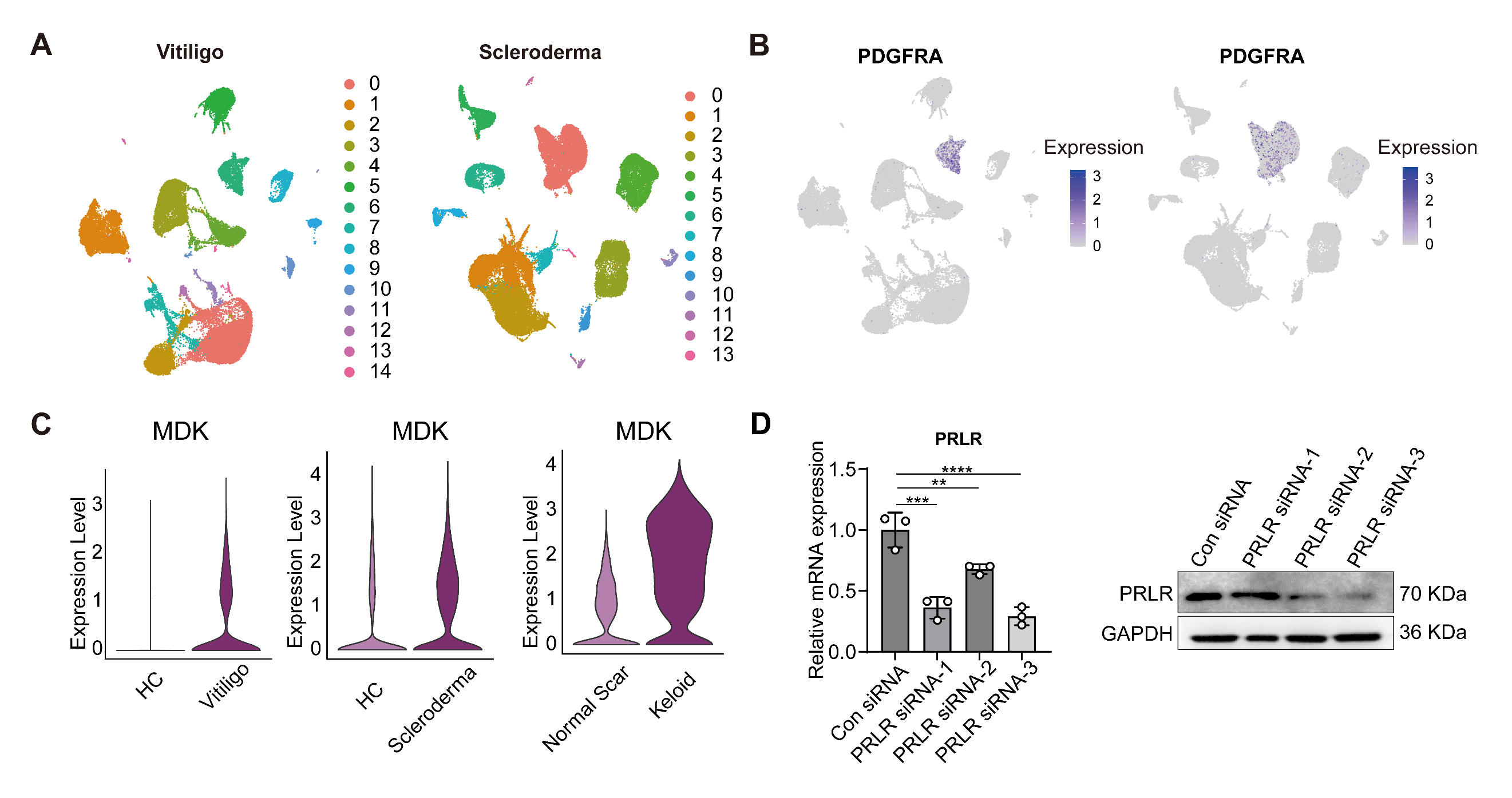


**Figure S7.** Elevated levels of MDK in dermal fibroblasts across several different psychosomatic skin disorders, related to Figure 5. A) UMAP embeddings of skin cells from vitiligo and scleroderma from public datasets. B) Scatter plot of *PDGFRA*^+^ fibroblasts in each skin condition. C) Violin plot of MDK expression in vitiligo, scleroderma, and keloid *vs*. respective normal controls from original datasets. D) Identification of PRLR knockdown efficiency by siRNAs in primary fibroblasts via qRT-PCR (left panel) and Western blot (right panel). PRLR siRNA3 was deployed to conduct subsequent experiments (*n* = 3). All results are shown as the means±SD. ^∗∗^*p* < 0.01, ^∗∗∗^ *p* < 0.001, ^∗∗∗∗^ *p* < 0.0001 (one-way ANOVA). HC: healthy control; MDK: midkine, PN: psoriatic non-lesional skin, PP: psoriasis skin.


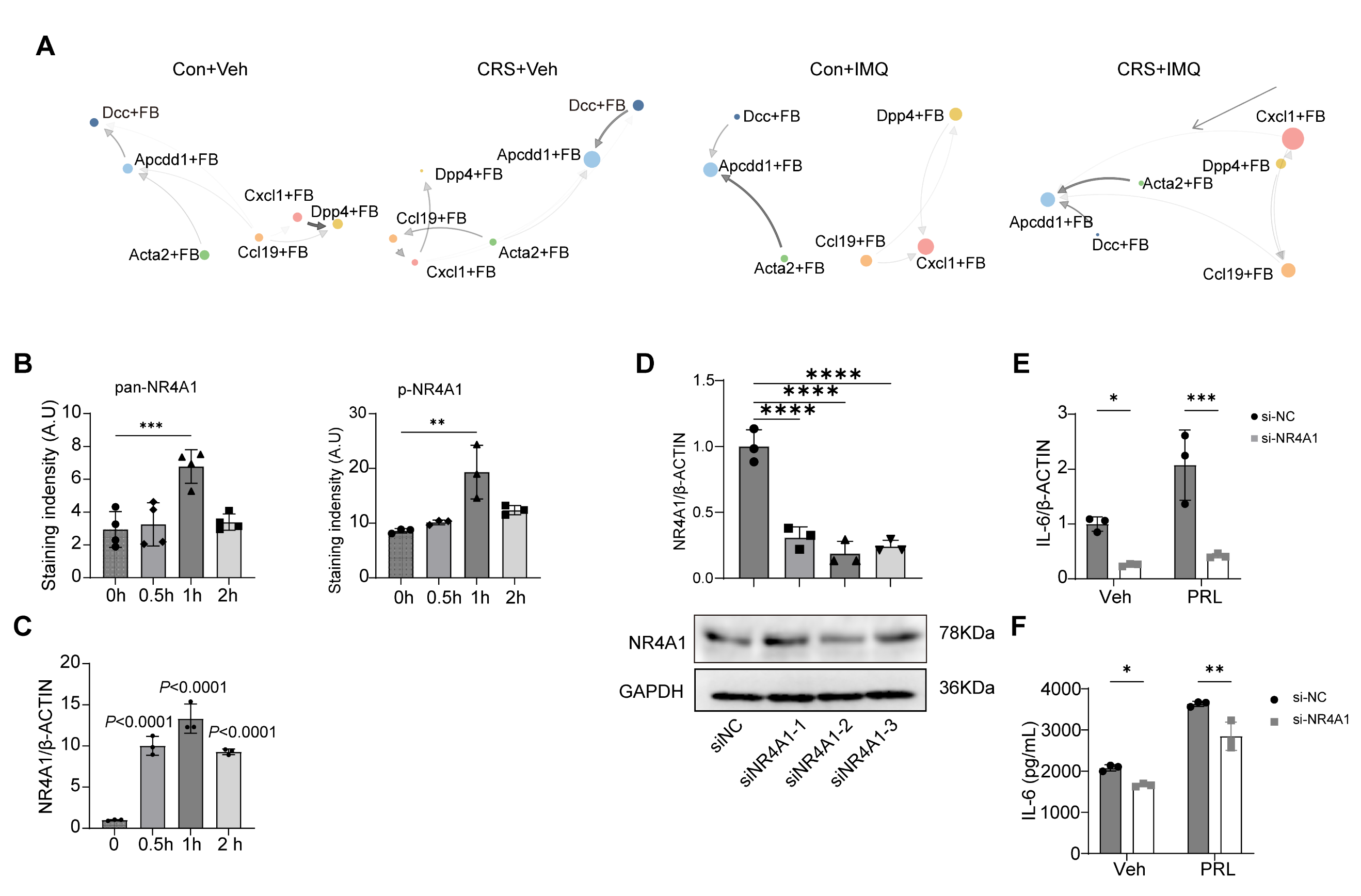


**Figure S8.** Prolactin activates immune response of dermal fibroblasts via NR4A1, related to Figure 6. A) State graph showing fibroblast subtype transitions from distinct mouse groups. B) Evaluation of mean fluorescence intensity of whole-cell (left) and nuclear (right) NR4A1 expression in PRL-stimulated fibroblasts (*n* = 3~4). C) Relative gene expression of NR4A1 in fibroblasts upon PRL stimuli (*n* = 3). D) Identification of NR4A1 knockdown efficiency by siRNAs in primary fibroblasts via qRT-PCR (upper panel) and Western blot (lower panel) (*n* = 3). NR4A1 siRNA2 was deployed to conduct subsequent experiments. E) Relative gene expression of IL-6 in fibroblasts upon PRL stimuli after siNR4A1 transfection (*n* = 3). F) Detection of IL-6 by ELISA using conditioned medium collected from fibroblasts with and without PRL stimuli (*n* = 3). All results are shown as the means±SD. ^∗^*p* < 0.05, ^∗∗^*p* < 0.01, ^∗∗∗^*p* < 0.001, ^∗∗∗∗^*p* < 0.0001 (B, C and D: one-way ANOVA, E and F: two-way ANOVA). A.U: Arbitrary Unit; Con: control; CRS: chronic restraint stress; ELISA: enzyme-linked immunosorbent assay; IMQ: imiquimod; PRL: prolactin; Veh: vehicle.


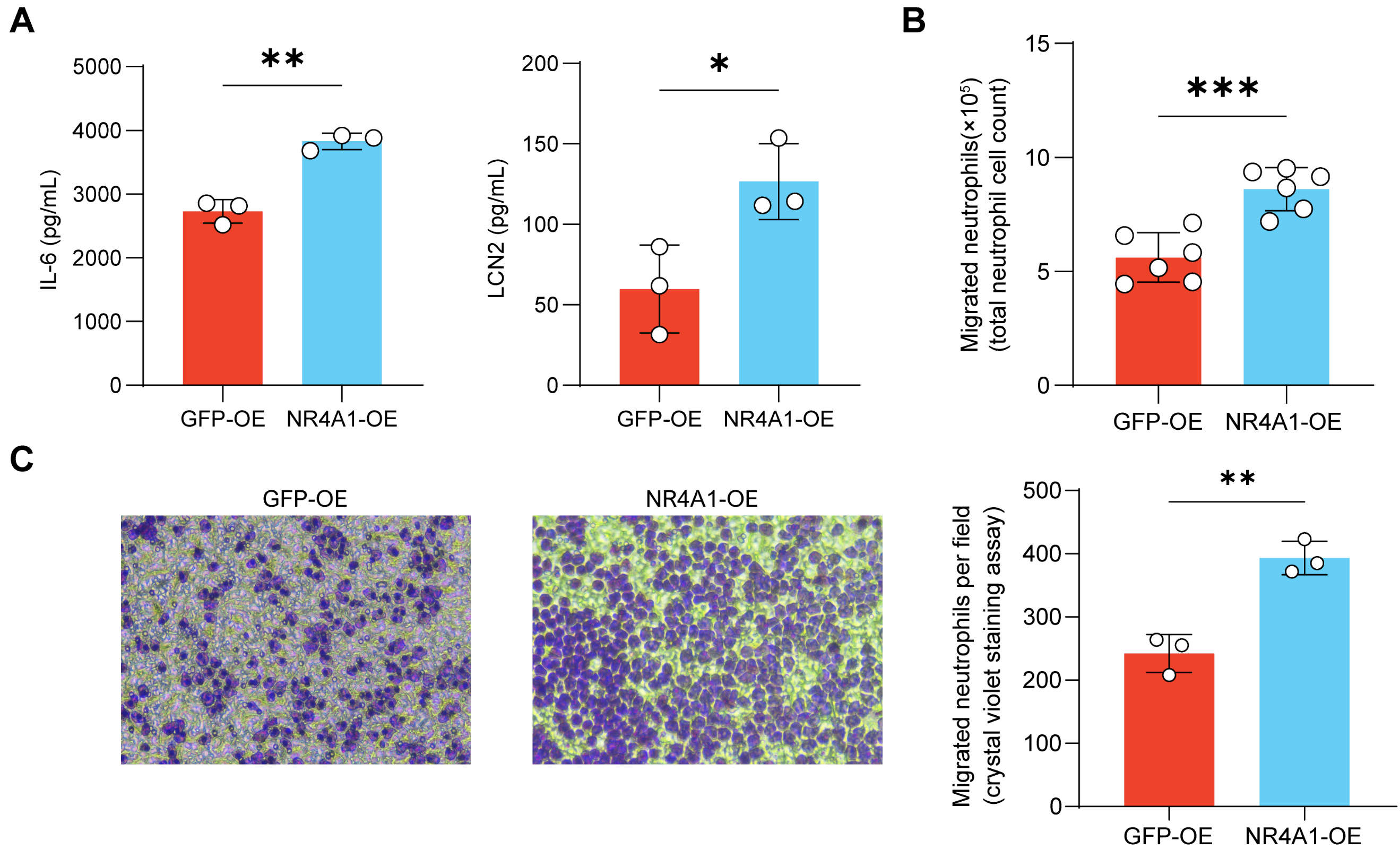


**Figure S9.** Overexpression of NR4A1 leads to the release of psoriasis-associated molecules in fibroblasts, related to Figure 7. A) Detection of inflammatory molecules by ELISA using conditioned medium collected from fibroblasts with and without NR4A1 overexpression (*n* = 3). B) The count of migration of neutrophils toward conditioned medium collected from fibroblasts with and without NR4A1 overexpression. C) The results of crystal violet staining for the lower transwell membrane. Images are representative of at least three time experiments. All results are shown as the means±SD. ^∗^*p* < 0.05, ^∗∗^*p* < 0.01, ^∗∗∗^*p* < 0.001 (unpaired Student’s *t* test). ELISA: enzyme-linked immunosorbent assay.
